# Fabrication of 6-gingerol, doxorubicin and alginate hydroxyapatite into a bio-compatible formulation: enhanced anti-proliferative effect on breast and liver cancer cells

**DOI:** 10.1186/s13065-018-0482-6

**Published:** 2018-11-23

**Authors:** Danushika C. Manatunga, Rohini M. de Silva, K. M. Nalin de Silva, Dulharie T. Wijeratne, Gathsaurie Neelika Malavige, Gareth Williams

**Affiliations:** 1 Department of Chemistry, University of Colombo, Colombo, 00300 Sri Lanka; 2 Sri Lanka Institute of Nanotechnology (SLINTEC), Nanotechnology & Science Park, Mahenwatte, Pitipana, Homagama, 10206 Sri Lanka; 3 Centre for Dengue Research, Department of Microbiology, Faculty of Medical Sciences, University of Sri Jayewardenepura, Nugegoda, 10250 Sri Lanka; 4 UCL School of Pharmacy, University College London, 29-39 Brunswick Square, London, WC1N 1AX UK

**Keywords:** Hydroxyapatite, 6-Gingerol, Doxorubicin, MCF-7, HEpG2

## Abstract

Ample attention has been devoted to the construction of anti-cancer drug delivery systems with increased stability, and controlled and targeted delivery, minimizing toxic effects. In this study we have designed a magnetically attractive hydroxyapatite (m-HAP) based alginate polymer bound nanocarrier to perform targeted, controlled and pH sensitive drug release of 6-gingerol, doxorubicin, and their combination, preferably at low pH environments (pH 5.3). They have exhibited higher encapsulation efficiency which is in the range of 97.4–98.9% for both 6-gingerol and doxorubicin molecules whereas the co-loading has accounted for a value of 81.87 ± 0.32%. Cell proliferation assays, fluorescence imaging and flow cytometric analysis, demonstrated the remarkable time and dose responsive anti-proliferative effect of drug loaded nanoparticles on MCF-7 cells and HEpG2 cells compared with their neat counter parts. Also, these systems have exhibited significantly reduced toxic effects on non-targeted, non-cancerous cells in contrast to the excellent ability to selectively kill cancerous cells. This study has suggested that this HAP based system is a versatile carrier capable of loading various drug molecules, ultimately producing a profound anti-proliferative effect.

**Electronic supplementary material:**

The online version of this article (10.1186/s13065-018-0482-6) contains supplementary material, which is available to authorized users.

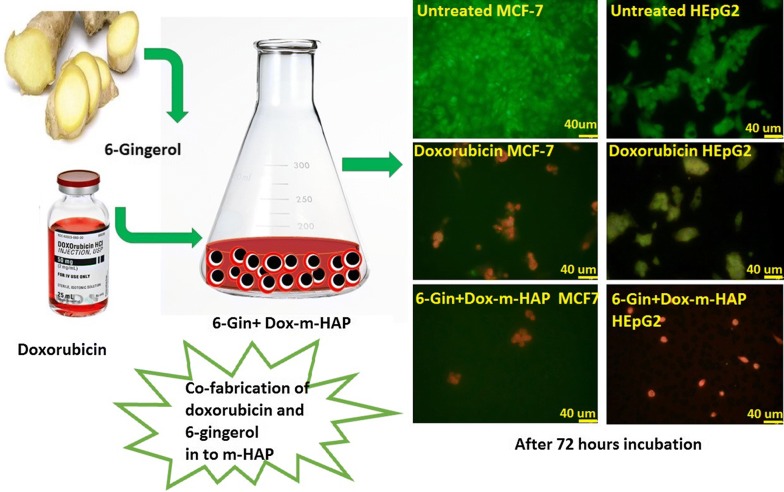

## Introduction

Doxorubicin is an extensively used first line chemotherapeutic [1, 2] with an excellent effectiveness over a range of cancer types including breast cancer and liver cancer [3–7]. It is an anthracycline which exerts its anti-proliferation effect by intercalating with double stranded DNA, which could in turn arrest cell division and expression of vital proteins, and ultimately lead to cell death [4, 5]. However, later on it was observed that this particular drug is heavily associated with cardiotoxicity, neurotoxicity, myelosuppression, non-targeted killing of normal or healthy cells, and the development of multi drug resistance (MDR), which has restricted its clinical efficacy and given rise to the recurrence of the cancers [8–11]. It has also been observed that the conjugation of doxorubicin with nanoparticulate systems such as superparamagnetic iron oxide nanoparticles would be an ideal approach to minimize the MDR while leading to enhanced cytotoxic effect over the drug resistant cancer cells [12, 13].

In addition, as a replacement approach for doxorubicin, the use of natural products as anti-cancer and cancer preventive agents has gained much attention over the past 30 years [14]. In this context, plant derived phytochemicals are preferred as they are generally less toxic and well tolerated by normal cells. These compounds generally contain a pool of active compounds such as alkaloids, phenolics, tannins and flavonoids with very high activity, including anti-oxidant, anti-inflammatory, anti-angiogenic, anti-microbial, anti-cancer activity [15]. Curcumin, gingerol, β-carotene, quercetin and linamarine are some of the commonly investigated compounds of plant extracts that are very effective and heavily investigated for the safer development of anti-cancer drugs [16–18].

6-Gingerol is a polyphenolic active ingredient of the ginger rhizome, *Zingiber officinale* [19, 20], which is capable of reducing the growth of many cancer types [17, 21, 22]. 6-Gingerol can interfere with number of cell signaling pathways that control the balance between the cell apoptosis and proliferation [23]. These beneficial effects have been mainly assessed for breast cancer and in liver carcinoma [19, 24]. Moreover, it has also shown anti-microbial, anti-viral, cardio-protective, anti-hyperglycemic, anti-lipidemic and immunomodulatory effects [25–27].

Nevertheless, 6-gingerol has various drawbacks such as temperature, pH, and oxygen sensitivity, light instability, and poor aqueous solubility, hindering its potential applicability [28, 29]. Therefore, the development of drug carrier systems for the safer delivery of 6-gingerol in a targeted and controlled manner is highly essential. Therefore, attention has been devoted to the development of a nanoparticle based delivery for these compounds [30]. However, the use of carriers for the delivery of 6-gingerol is limited to a few studies [20, 26, 28, 31].

The co-delivery approach of 6-gingerol with toxic chemotherapeutics such as doxorubicin and cis-platin is another area of 6-gingerol utilization as it could synergistically act along with these drug molecules due its chemo preventive and chemo sensitive properties [20]. 6-Gingerol has been very effective in the elimination of the problem of MDR, seen with many chemotherapeutics [32]. Furthermore, the synergistic effect of 6-gingerol on neuroprotective, hepatoprotective, and anti-emetic properties has been exhibited when co-administering with doxorubicin [25, 32–37].

Nevertheless, it is worth noticing that the use nanoparticle based targeted and controlled drug delivery carriers for the dual loading of doxorubicin and 6-gingerol and enhancing their properties is not reported elsewhere. Therefore, in this study we have attempted to use a novel magnetic hydroxyapatite (m-HAP) nanoparticle system as an effective drug carrier for the controlled and pH sensitive delivery of 6-gingerol, doxorubicin and the dual drugs to inhibit the proliferation of breast and liver carcinoma cells targeting the development of a universal type drug carrier.

## Materials and methods

### Materials

6-Gingerol (> 98.0%, HPLC), Doxorubicin hydrochloride (98.0–102.0%, HPLC), calcium nitrate tetrahydrate (Ca(NO_3_)_2_·4H_2_O, 99%, ACS), diammonium hydrogen phosphate ((NH_4_)_2_HPO_4_, > 99.0%), ammonium iron(II) sulfate hexahydrate ((NH4)_2_Fe(SO_4_)_2_·6H_2_O, 99.0%, ACS), ammonium iron(III) sulfate dodecahydrate (NH_4_Fe(SO_4_)_2_·12H_2_O, 99.0%, ACS), ethanol (EtOH, > 99.8%, HPLC), methanol anhydrous (MeOH, 99.8%), alginic acid sodium salt (NaAlg, low viscosity), Cetyltrimethyl ammonium bromide (CTAB, > 98%) and TWEEN^®^80 (Viscous liquid), and ammonium hydroxide solution (puriss. p.a., 25% NH_3_ in H_2_O) were purchased from Sigma Aldrich, Bangalore, India. Polyethylene glycol 200 (PEG 200) was purchased from Merck Millipore Corporation, Darmstadt, Germany. Snakeskin dialysis tubing (MWCO 3.5 kDa) was purchased Thermo Fisher, Bangalore, India.

### Cell lines and reagents

MCF-7 breast carcinoma cell line and HEpG2 hepatocellular carcinoma cell line were purchased from ECACC (Salisbury, UK) and cultured in complete DMEM (Gibco, UK). The DMEM medium was supplemented with 10% fetal bovine serum (FBS, Gibco, USA), 100 U/mL of penicillin and 100 μg/mL of streptomycin, 1% 200 mM l-glutamine (Gibco, USA) and 1% non-essential amino acids (NEAA, 100×, Gibco, USA) whereas the RPMI medium (RPMI 1640, Gibco, UK), supplemented with 10% FBS, 1% l-glutamine, and 1% penicillin/streptomycin, was used to culture HEpG2 cells. Both cell cultures were maintained at 37 °C in a humidified 5% CO_2_ atmosphere.

To assess the effect of these nanoparticles on non-targeted cells, African Green monkey kidney epithelial cell line, Vero (ATCC, USA) was purchased and grown in DMEM medium containing 10% FBS, 1% penicillin/streptomycin, 1% l-glutamine, 1% NEAA and 1% 1 M NaHCO_3_ under standard cell culture conditions. Passaging of all three cell lines was carried out every 3–4 days using 0.05% Trypsin EDTA.

### Preparation of magnetic HAP (m-HAP) and in vitro loading of drug molecules

Briefly, PEG coated IONPs were prepared using 25.0 mL of 0.1 M iron precursor solutions with 2:1 (Fe^3+^:Fe^2+^) which were later functionalized with sodium alginate polymer molecules (0.500 g of PEG coated IONPs mixed with 40% w/v of sodium alginate). HAp nanoparticles were allowed to be generated as a coating on the alginate-IONPs to obtain magnetic HAP as specified in our previous work [38]. 6-Gingerol and doxorubicin were selected as the potential anti-cancer drug and a positive control respectively. Their individual loading and the combinational loading was carried out using m-HAP as a drug carrier material. The 6-gingerol loading procedure was similar to the process specified by our group in previous work [38] and the obtained product is labelled as 6-Gin-m-HAP.

In addition, the loading of doxorubicin onto m-HAP involved the incubation of 0.06 g/mL m-HAP solution with 66.67 mL of 25 ppm aqueous doxorubicin. HCl solution provided with mild stirring for 17 h at 37 °C. Doxorubicin loaded m-HAP (Dox-m-HAP) was magnetically separated, and the unbound doxorubicin content was determined via fluorescence spectroscopy [39], λ_excitation_ at 467 nm and λ_emission_ at 589 nm, HORIBA fluorescence spectrophotometer).

For the dual loading of 6-gingerol and doxorubicin (6-Gin + Dox-m-HAP), m-HAP loaded with 6-gingerol (23.0 mg of 6-gingerol dissolved in methanol) was separated from the original solution and incubated with the 25 ppm doxorubicin solution for 17 h at 37 °C.

To assess the amount of 6-gingerol loaded into 6-Gin-m-HAP and 6-Gin + Dox-m-HAP, an analysis of the samples was carried out using UV Visible spectroscopy (Grant XUB5, Grant Instruments) at 291 nm which corresponds to the λ_max_ of desorbed 6-gingerol in methanol medium [38].

From the results obtained for the loaded 6-gingerol and doxorubicin, from the UV measurements and fluorescence spectroscopy, respectively, the two important parameters of the drug carrier, which are the loading capacity and the loading efficiency were calculated [40].

To measure the drug release from these formulations, 10.0 mg of the drug loaded nanoparticles were inserted into a dialysis bag (MWCO 3500) and incubated in 20 mL of PBS buffer (pH 7.4, PBS:MeOH = 9:1) and acetate buffer (pH 5.3, Ace:MeOH = 9:1) at 37 °C provided with mild shaking (80 rpm) over a period of time. At regular time intervals, 0.5 mL aliquots of the sample were withdrawn from the solution and replaced with the fresh buffer. The amount of released 6-gingerol and doxorubicin was analyzed according to the procedure specified above. The cumulative drug release in each drug system was calculated. All the studies were carried out in triplicate in three individual experiments.

### Characterization of m-HAP, 6-Gin-m-HAP, Dox-m-HAP, 6-Gin + Dox-m-HAP

The size and the morphology of the m-HAP, 6-Gin-m-HAP, Dox-m-HAP and 6-Gin + Dox-m-HAP were acquired using a transmission electron microscope (TEM, JEOL JEM-2010 High resolution transmission electron microscope, Japan) operating at 80 kV. The different functional groups of the carrier and the drug-carrier molecules were identified using Fourier transform infra-red (FT-IR) spectroscopy (Bruker Vertex 80, Germany) via the diffuse reflectance mode, within the spectral range 400–4000 cm^−1^. Further, the interaction of the drug molecules with the carrier was studied using X-ray photoelectron spectroscopic (XPS) analysis (a K-alpha instrument, Thermo Scientific, East Grinsted, UK, equipped with a monochromated Al Kα X-ray source was used with a pass energy of 40 eV and step size of 0.1 eV). Spectra were processed using the CasaXPS software (Casa Software Ltd., Teignmouth, UK).

### In-vitro cytotoxicity assessment

The in vitro cytotoxicity of different formulations (m-HAP, 6-Gin-m-HAP, Dox-m-HAP and, 6-Gin + Dox-m-HAP) on MCF-7 breast cancer cells and HEpG2 liver cancer cells was assessed using WST-1 cell proliferation detection assay [41]. Briefly, cells were seeded in 96-well plates (Greiner CELLSTAR^®^) at a density of 3 × 10^3^ cells/well [42–44] and they were cultured overnight in the respective media under standard cell culture conditions. The cells were then incubated with different concentrations of drugs and nanoparticles for 24, 48 and 72 h. Subsequently, 10 µL of the WST-1 solution (Abcam, ab155902, UK) were added to each well, and the cells were incubated for 0.5–4 h in standard culture conditions without the removal of the media. Later, absorbance values were recorded with an ELISA plate reader (MPScreen MR-96A) at 450 nm with a reference wavelength at 630 nm. Experiments were performed in triplicate in three individual experiments. The percentage inhibition was obtained as given in the following equation (Eq. 1) [45].

1 $$Percentage\,cell \,inhibition \;\left( \% \right) = 1 - \frac{{A_{cells\,+\,nanoparticles } - A_{blank} }}{{A_{cells} - A_{blank} }} \times 100\%$$

A_cell_ and A_(cells+ nanoparticles)_ are the absorbance values for the untreated cells and those treated with the nanoparticles, respectively. A_blank_ is the absorbance of the medium only. Triplicate data from three individual experiments were used to calculate the inhibitory concentration (IC)50 using GraphPad Prism 5 software (GraphPad Software Version 7.02, USA).

### In-vitro cellular uptake studies

Cells were seeded in 8 well chamber slides (Nunc^®^ Lab-Tek^®^ Chamber Slide™) with a density of 2.5 × 10^4^ cells/well [42] overnight under standard cell culture conditions. The cells were then treated with 1C_50_ values of 6-Gin-m-HAP, Dox-m-HAP and 6-Gin + Dox-m-HAP for 24, 48 and 72 h. All the experiments were carried out in triplicate, and after each incubation the cells were washed twice with cold PBS and then fixed with 3.7% paraformaldehyde S (VWR, UK) for 15 min prior to staining. The fixed cells were washed and stained with AO/EB (100 µg/mL) dual staining for 10 min under dark conditions [43]. Similarly, for Hoechst staining the fixed cells were washed and treated with 5 µg/mL Hoechst (Thermo Fisher Scientific, Life Technologies) for 15 min [44]. After each incubation the stained cells were visualized under the fluorescence microscope (Olympus, FSX100).

### Flowcytometric analysis of apoptotic induction

A quantitative measurement on apoptosis was obtained via flow cytometric analysis which required Annexin V APC and Zombie green dual staining protocol of cells [46]. The cells were seeded at a density of 1.5 × 10^5^ cells/well in a 24 cell well plate overnight under standard cell culture conditions. The medium was replaced with media containing the nanoparticles corresponding to the IC_50_ values of each system. The incubation was continued for 18 h. Then the cells were trypsinized, centrifuged, washed and the pellet was treated with 0.5 µL of Zombie green for 30 min at room temperature. This was then washed with 2% FBS in PBS and subjected to Annexin V staining (195 µL of Annexin V binding buffer and 5 µL Annexin V APC) for 10 min at room temperature. All the steps were carried out under dark conditions. At the end of the staining the cells were immediately analyzed using flow cytometer (Guava-easyCyte flowcytometser, Merck). The cells devoid of nanoparticles and treated only with media served as the control. All the samples were run in triplicate. The data were analyzed by FCS express version 4 (denovo software).

### Evaluation of the effect on non-cancerous mammalian cells

It is also important to detect whether these nanoparticles could selectively act on cancerous cells, providing a least or no effect on the non-targeted cells during the delivery. For this purpose, the cytotoxicity of bare nanoparticles and the drug loaded nanoparticles on a non-cancerous, epithelial cell line, i.e., Vero cell line, was evaluated [47, 48]. The cells were seeded at a cell density of 1 × 10^3^ cells/well in a 96 well plate and on the following day they were treated with a series of different concentrations of nanoparticles and further incubated for another 24 h. At the end of the incubation, cell viability was assessed via the WST-1 cell viability assessment assay as specified earlier. All the samples were analyzed in triplicate.

### Statistical analysis

The data were presented as the mean ± SEM. One-way analysis (ANOVA) of variance was used to determine statistical significance of the cumulative release rate and cell viability followed by Tukey–Kramer post hoc test analysis of variance. *P* values < 0.05 were considered statistically significant. All statistical analyses were performed using SPSS version 19 (IBM Corporation, Armonk, NY, USA).

## Results and discussion

### Morphological characterization of 6-Gin-m-HAP, Dox-m-HAP, and 6-Gin + Dox-m-HAP

**Fig. 1:**
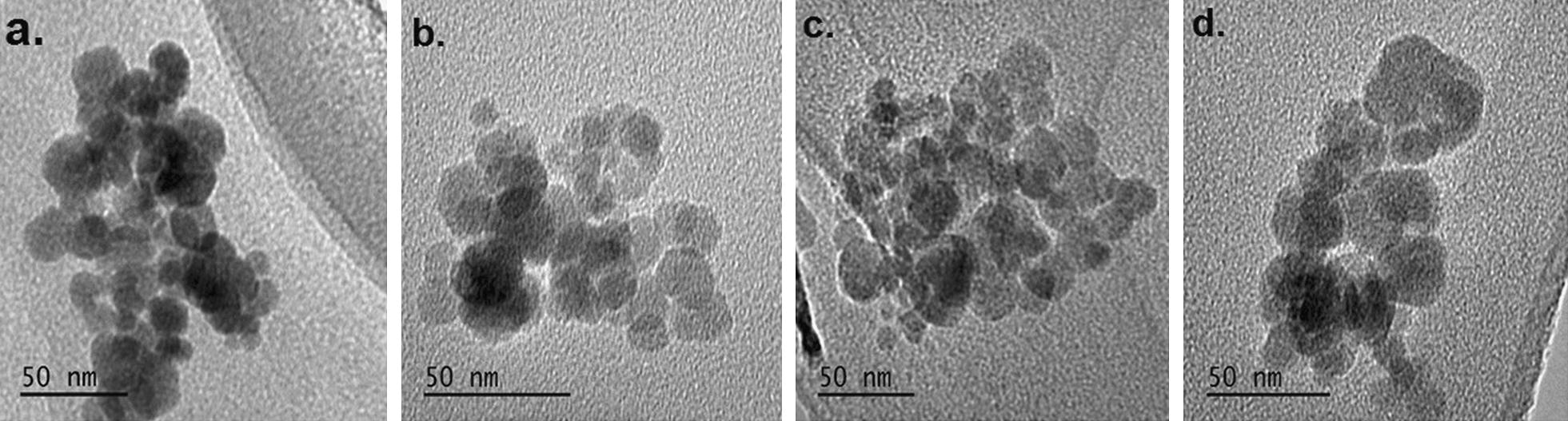
TEM images of **a** neat drug carrier (m-HAP), **b** 6-Gin-M-HAP, **c** Dox-m-HAP and **d** 6-Gin + Dox-m-HAP

According to TEM images in Fig. 1, neat nanoparticles (m-HAP) have sizes ranging from 10 to 20 nm; with drug loading the nanoparticles tend to increase in size. It is observed that the 6-Gin-M-HAP, Dox-m-HAP and 6-Gin + Dox-m-HAP nanoparticles are in the sizes of 21.6 ± 1.5 nm, 28.2 ± 2.1 nm and 32.1 ± 4.3 nm respectively. It can be seen that the presence of 6-gingerol or doxorubicin has given rise to an enlarged agglomerated nature (Fig. 1).

#### Surface functionalization characterization via FT-IR and XPS studies

**Fig. 2:**
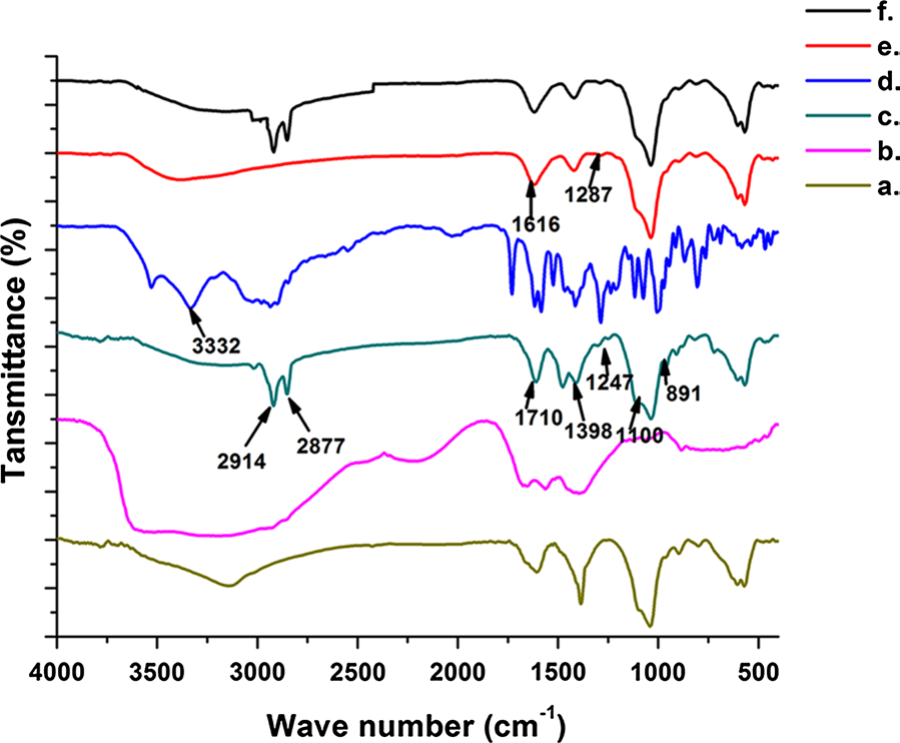
FT-IR characterization of **a** m-HAP, **b** neat 6-gingerol, **c** 6-Gin-m-HAP, **d** neat doxorubicin, **e** Dox-m-HAP, **f** 6-Gin + Dox-m-HAP

FT-IR spectra obtained for the four systems are given in Fig. 2, where Fig. 2a, b display the FT-IR spectra for neat drug carrier and the neat 6-gingerol, respectively. After loading of 6-gingerol onto m-HAP (Fig. 2c), peaks corresponding to –CH_2_ stretching are clearly appearing at 2914 cm^−1^ and 2877 cm^−1^. The rest of the peaks resemble the m-HAP spectrum with some additional peaks [49]: a small hump at 1719 cm^−1^ corresponding to –C=O stretching [26], and a few bands at 1398 cm^−1^, 1247 cm^−1^, 1110 cm^−1^, and 891 cm^−1^ corresponding to aromatic C–H in-plane deforming and stretching, –C–O–C stretching, and –C–O stretching of –C–O–H bonds, respectively [50, 51].

The disappearance of the broad –NH_2_ stretching band at 3332 cm^−1^ of doxorubicin (Fig. 2d) in Dox-m-HAP (Fig. 2e) is in good agreement with the doxorubicin interacting with the drug carrier [52]. In Fig. 2e the appearance of a band at 1616 cm^−1^ [53] and a weak band at 1287 cm^−1^ corresponding to carbonyl and –C–O–C– stretching vibration respectively of the doxorubicin further confirmed the incorporation of doxorubicin into the nanoparticles [54].

When both 6-gingerol and doxorubicin were co-loaded to the m-HAP (Fig. 2f), most of the bands in the fingerprint region of doxorubicin and 6-gingerol appear weak, due to the restriction of bond vibration when they are blended together in nanoparticles [55].

**Fig. 3:**
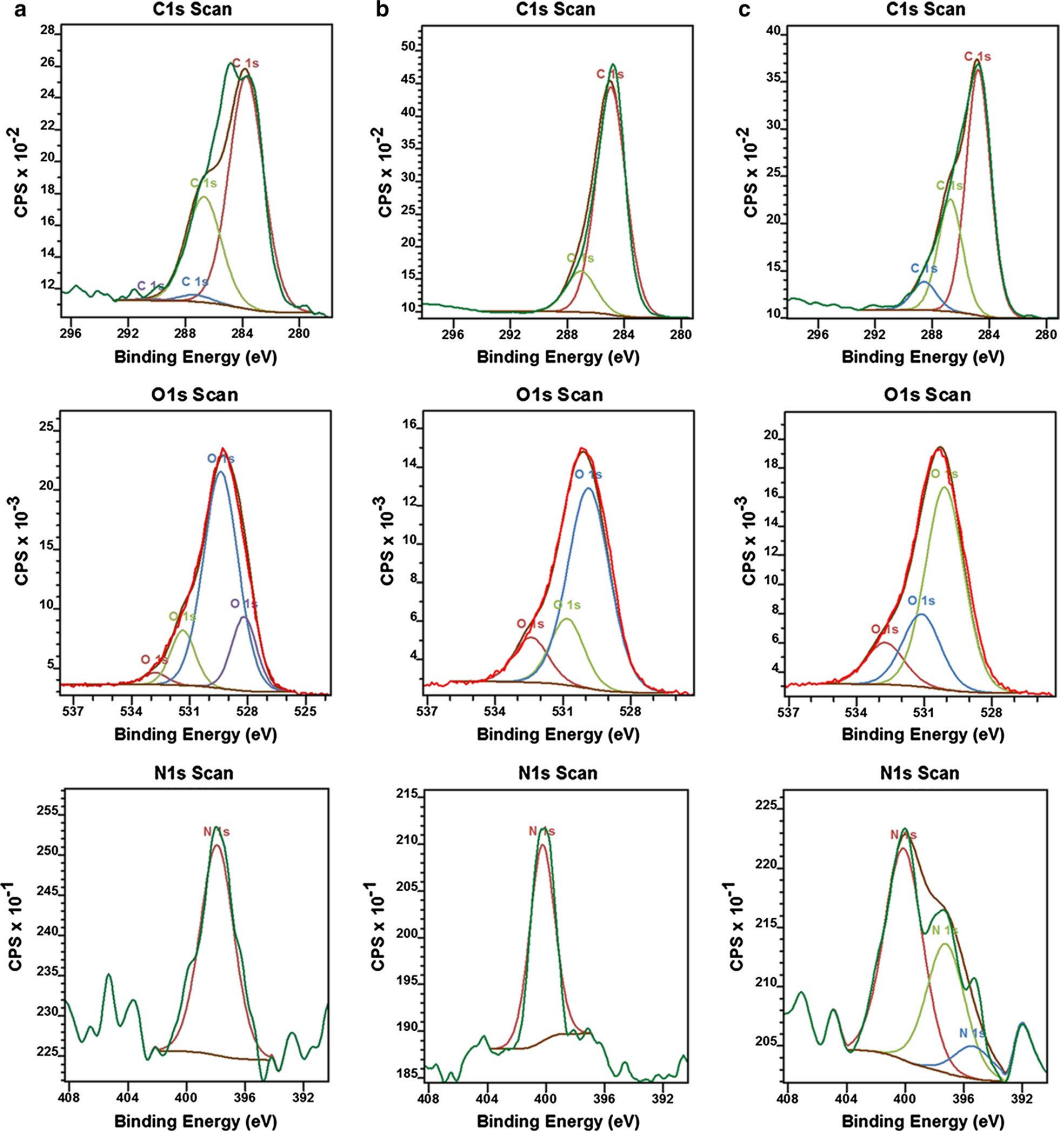
XPS analysis of drug loaded nanoparticle systems with the corresponding binding energy spectra for C1s, O1s and N1s: **a** Dox-m-HAP, **b** 6-Gin-m-HAP and **c** 6-Gin + Dox-m-HAP

In Fig. 3a–c, the XPS data of C1s, O1s and N1s obtained for Dox-m-HAP, 6-Gin-m-HAP and 6-Gin + Dox-m-HAP are presented. In Dox-m-HAP (Fig. 2a) system, the peak at 283.72 eV would arise due to the –C=C/C–C [56] of doxorubicin with slight shift due to the cation–π interactions. The peak appearing at 286.69 eV could be due to the –C–N bonds resulting from doxorubicin [57] or –C–O–C– bonds of alginate or doxorubicin [58]. In addition, C1s peaks at 287.26 eV and 290.36 eV would appear due to –C–O–H of alginate [59] and O=C– bonds of doxorubicin/alginate respectively [60]. Regarding the O1 s spectrum, the peak at 532.79 eV could appear from the O=C– bonds of doxorubicin or alginate [59], or the adsorbed water [61]. Furthermore, the peaks appearing at 531.35 eV and 529.39 eV would be accounted for as the –O– of HAP [62, 63] or the O=C–C– of doxorubicin [64]. Evidence for the presence of –C–N bond of doxorubicin was seen in the N 1 s spectrum and it could be expected that the decrease of the peak position by few eVs would arise due to the binding of doxorubicin to Ca^2+^ due to the transfer of the electron density of N–Ca^2+^ ions, similar to what was observed with the Au–N interaction in previous studies [57].

In system 6-Gin-m-HAP (Fig. 3b), a C1s peak at 284.94 eV is also present due to –C=C– binding energy [56] or due to –C–H bonds of CTAB molecules [65]. However, a slight increase of the binding energy can be accounted for by the reduction of electron density by the electron attracting groups around carbon atoms. The presence of gingerol is also indicated by the O1s binding energy peak corresponding to O=C– bonds at 532.41 eV [59]. Further, presence of quaternary amines of CTAB will lead to N1s peak at 400.26 eV [65, 66] with a shift to indicate electrostatic interaction with the alginates.

In the 6-Gin + dox-m-HAP system, C1s peaks at 288.13 eV, 286.74 eV, and 288.13 eV suggest the presence of O=C– of doxorubicin [60], C–N of doxorubicin/CTAB [65, 67] and C=O of gingerol/alginate, respectively [58, 59]. The presence of doxorubicin and gingerol was further indicated by the O1s peak at 532.73 eV corresponding to O=C–O [58, 59]. Evidence for the presence of various nitrogen environments was provided by N 1 s peaks appearing at 400.10 eV, 397.23 eV, and 395.37 eV corresponding to –C–N of CTAB [65, 67], –C–N of doxorubicin [64, 67] and the formation of –N=N– bond (NIST) between the nanoparticle bound cross-linked doxorubicin molecules. The XPS analysis of neat drug carrier (m-HAP) is given in Additional file 1: Fig. S1.

### Assessment of the drug loading ability of m-HAP drug carrier

The drug loading ability of m-HAP was also quantified by measuring the drug loading capacity (DL) and the drug encapsulation efficiency (EE) for each system. The resulting DL values and EE values are given in the Table 1, and reveal that the DL capacity has increased in 6-Gin + Dox-m-HAP, where they are co-fabricated together, compared to the 6-Gin-m-HAP system. This could be due to the favorable interactions among 6-gingerol and doxorubicin molecules.

**Table 1:** Drug loading capacities and encapsulation efficiencies of drug loaded into m-HAP

System	DL (%)	EE (%)
6-Gin-m-HAP system	3.77 ± 0.55	98.8 ± 0.05
Dox-m-HAP system	23.0 ± 0.33	97.4 ± 0.12
6-Gin + Dox-m-HAP system	20.0 ± 0.12	81.9 ± 0.32

### In vitro drug releasing studies of 6-Gin-m-HAP, Dox-m-HAP and 6-Gin + Dox-m-HAP

The in vitro drug release profiles of loaded drugs are given in Additional file 1: Fig. S2. In-contrast to the neat drugs which displayed a rapid and a complete release, when the drug was releasing from m-HAP the releasing pattern for both 6-gingerol and doxorubicin displayed a bi-phasic mode, of which the initial 1–6 h accounted for a burst release followed by a much slower, sustained release [68]. It was also noticeable that this release is preferred at low pH (pH 5.3) than at neutral pH, highlighting the pH sensitivity of the carrier, m-HAP [38]. The release of 6-gingerol, at 5.3 pH after an incubation period of 96 h, was 99.48 ± 0.70% for the singly loaded situation, while it was 99.46 ± 0.63% when co-loaded with doxorubicin, after an incubation period of 168 h.

As far as releasing doxorubicin from the carrier is concerned, a low drug release percentage of 49.37 ± 0.85% and 14.28 ± 0.54% were recorded when singly loaded and co-loaded with 6-gingerol, respectively. This could be attributed to strong interactions of doxorubicin with alginate, HAp and iron oxide such as electrostatic and van der Waals interactions, and H-bonding [69, 70]. Also the release of doxorubicin could be retarded due to the competition that would build up between the doxorubicin and 6-gingerol in the co-loaded situation.

### In-vitro cytotoxicity assessment

**Fig. 4:**
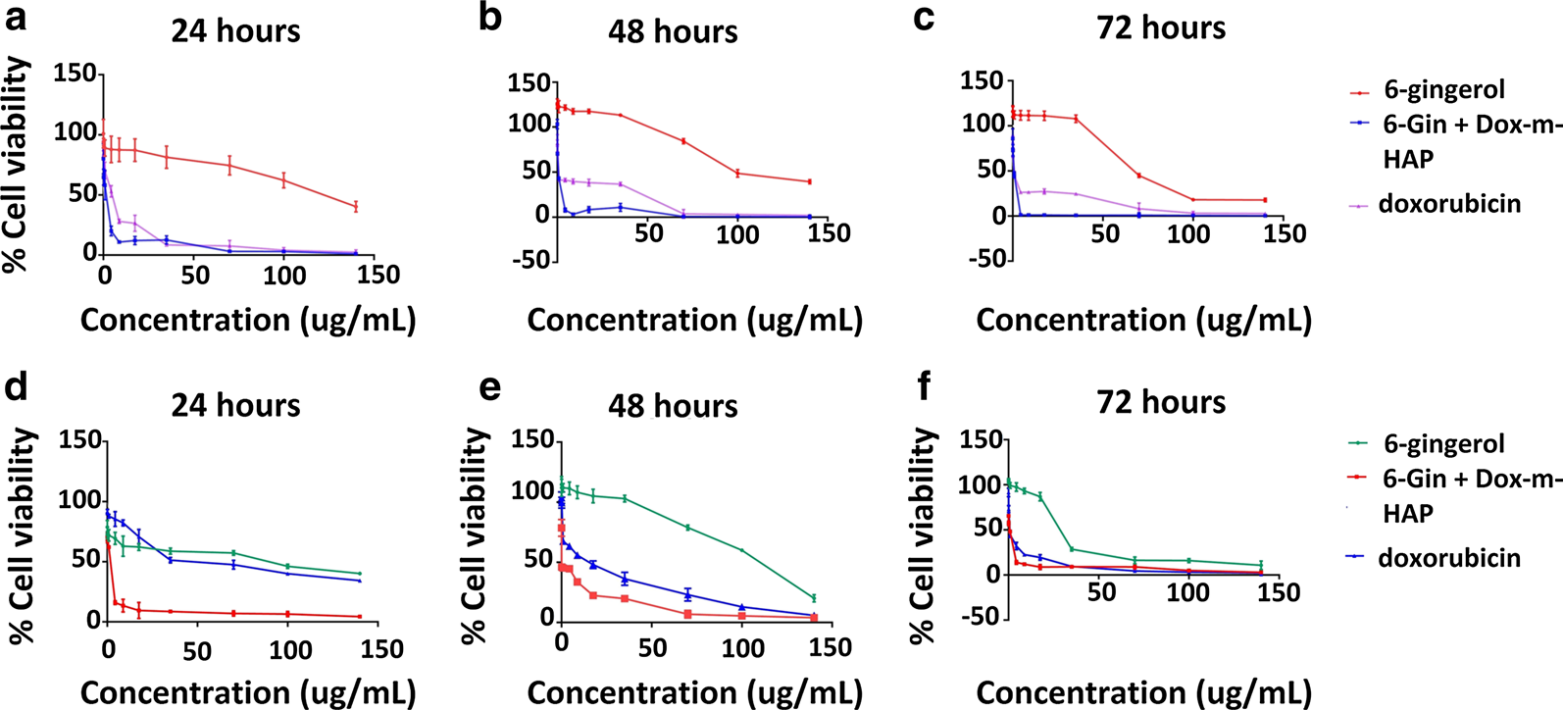
Dose response and time response curves of MCF-7 and HEpG2 cells treated with 6-gingerol, doxorubicin and 6-Gin + Dox-m-HAP. **a**–**c** Effect of these systems on MCF-7 cells over 24, 48 and 72 h. **d**–**f** Effect of these systems on HEpG2 cells over 24, 48 and 72 h. Results are given as mean ± SD, n = 3

In order to verify the anti-proliferative potential of these drug nano-conjugates, proliferation inhibition assays were conducted over three time points: 24, 48 and 72 h. The corresponding dose responsive and time responsive curves for 6-gingerol and doxorubicin systems over the two cell lines are given in Additional file 1: Figs. S3 and S4, whereas Fig. 4 show the time and dose response activity of the 6-Gin + Dox-m-HAP system on the same cell lines. The calculated IC_50_ values are given in Table 2.

**Table 2:** Corresponding IC_50_ values for each drug system at 24, 48 and 72 h incubation with cells

Drug system	IC50 on MCF-7 cells (µg/mL)			IC50 on HEpG2 cells (µg/mL)		
	24 h	48 h	72 h	24 h	48 h	72 h
m-HAP	Inactive	Inactive	Inactive	Inactive	Inactive	Inactive
6-Gingerol	150.5 ± 10.6	102.4 ± 2.9	67.4 ± 6.9	118.9 ± 8.2	115.0 ± 7.9	32.1 ± 7.4
6-Gin-m-HAP	2.25 ± 0.73	1.38 ± 0.30	0.81 ± 0.17	44.9 ± 7.5	24.4 ± 7.8	3.43 ± 2.60
Doxorubicin	2.96 ± 0.96	3.03 ± 0.96	1.09 ± 0.46	53.0 ± 0.2	14.9 ± 4.1	1.14 ± 0.31
Dox-m-HAP	0.53 ± 0.15	0.43 ± 0.15	0.16 ± 0.05	7.69 ± 1.73	7.28 ± 2.19	0.58 ± 0.24
6-Gin + Dox-m-HAP	0.58 ± 0.07	0.53 ± 0.12	0.45 ± 0.09	0.55 ± 0.19	0.47 ± 0.05	0.19 ± 0.06

In general, it is clear that neat doxorubicin and Dox-m-HAP are very potent in their activity, leading to very low IC_50_ values with respect to other systems acting on both MCF-7 and HEpG2 cells. However, there is a significant increase in activity when drugs are loaded onto m-HAP nanoparticles, in contrast to the neat drug. This could be due to the remarkable ability of the carrier molecules to penetrate the cell membranes and to extend the activity [71].

When consider the effect of the 6-Gin + Dox-m-HAP system on MCF-7 cells (Fig. 4a–c and Table 2), it is as active as the Dox-m-HAP system (Additional file 1: Fig. S3), while maintaining a better activity than 6-Gin-m-HAP, neat doxorubicin and neat 6-gingerol. The equal behavior of these two drug formulations suggests that the major effect is coming from the Dox-m-HAP system.

However, this system has been very promising against HEpG2 cells by having lowered IC_50_ values with respect to all the other drug systems (Fig. 4, Additional file 1: Fig. S4 and Table 2). This emphasizes that this combinational delivery system is more effective against HEpG2 cells than MCF-7 cells. This may result from the synergistic effect of those two compounds which will enhance the cytotoxic activity on cancer cells [32].

Nevertheless, this type of a combinational approach highlights the novelty of this work as there are no reports on the use of a nanoparticle based drug carrier for the co-delivery of both 6-gingerol and doxorubicin for the treatment of cancer, more specifically the treatment of liver and breast cancer.

### Fluorescence imaging of cellular uptake and damage

The IC_50_ value was used to assess the cell damage that is induced by the neat drug or the drug loaded nanoparticles on MCF-7 and HEpG2 cells for 24–72 h. The morphological and nuclear changes that take place, detected via a fluorescence staining protocol (i.e., use of Hoechst and AO/EB staining), indicated that the apoptosis induction ability of 6-gingerol has been enhanced by loading onto the m-HAP carrier (Additional file 1: Fig. S5a, b). However, these cells have displayed reduced volume, round shaped cells, and brighter nuclei with Hoechst [72], and bright yellow to red orange nuclei with AO/EB staining [73], confirming that a major proportion of cells are affected and have lost cellular integrity.

Likewise, when the doxorubicin system is considered (Additional file 1: Figs. S6a–c, S7a–c), Dox-m-HAP has been far superior to 6-Gin-m-HAP in exhibiting reduced remaining cell count with time. This could be due to the removal of dead cells during the staining procedure due to the loss of adherence. This is further demonstrated by the reduction of intensity of Hoechst stained cells.

**Fig. 5:**
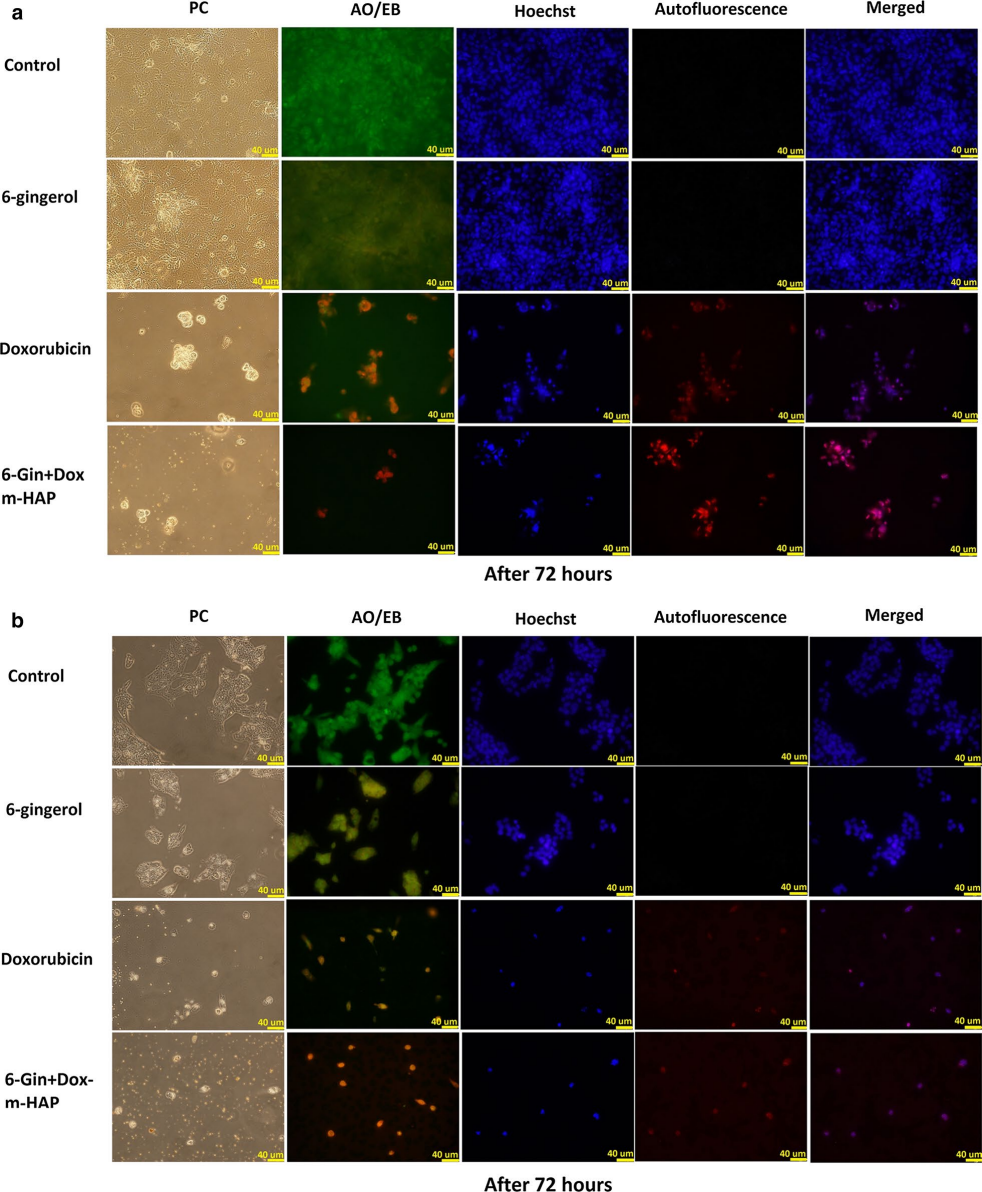
Phase contrast (PC) and fluorescence images obtained to assess the effect of 6-gingerol, doxorubicin and 6-Gin + Dox-m-HAP on **a** MCF-7 cells, **b** HEpG2 cells incubated for 72 h. Scale bar is 40 µm

A marked effect was also observed with the co-fabricated system indicating its enhanced activity over neat doxorubicin and 6-gingerol, with more of the cells undergoing apoptosis and loss of attachment, and thereby reducing the remaining cells (Fig. 5a, b and Additional file 1: Fig. S8a, b).

### Quantitative apoptotic detection via flow cytometry

**Fig. 6:**
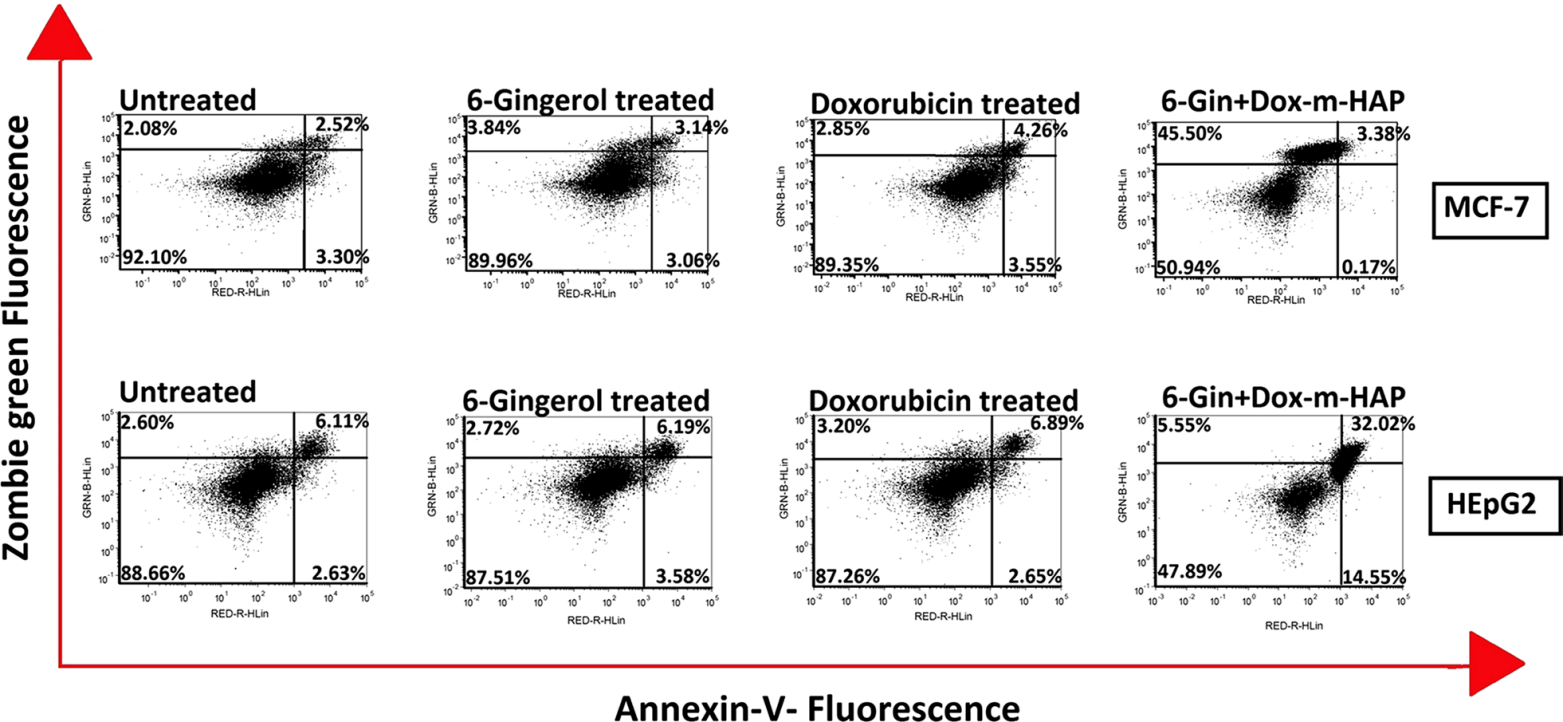
Flow cytometric analysis of apoptotic induction of MCF-7 and HEpG2 cells by 6-gingerol, doxorubicin and 6-Gin + Dox-m-HAP after staining with Annexin V (ANX) and Zombie green (ZGR) dyes. ANX^−^/ZGR^+^: necrotic or debris cells; ANX^+^/ZGR^+^: late apoptotic cells; ANX^−^/ZGR low: viable; ANX^+^/ZGR dim: apoptotic cells. Numbers in each quadrant represent the percentage of cells (data are given as mean ± SD of triplicate experiments)

In vitro anti-tumor activity of drug loaded nanoparticles was also quantitatively assessed by categorizing the cell population into different stages using flow cytometry. As shown in Fig. 6, a very few necrotic, debris or apoptotic cells could be detected in untreated cells where most of them remain viable. In contrast, when the cells are treated with free drug or the drug loaded nanoparticles, there is a clear shift of the cells from viable to apoptotic, late apoptotic and necrotic stages, decreasing the viable cell count. This is observed with doxorubicin, Dox-m-HAP, 6-Gin-m-HAP, and 6-Gin + Dox-m-HAP treated cells of both cell lines (Fig. 6, Additional file 1: Fig. S9a, b). It is clear that, with respect to the free drugs and singly loaded systems, the co-fabricated system (i.e., 6-Gin + Dox-m-HAP) has led to a higher percentage of apoptotic, late apoptotic and necrotic cells, amounting to 49.05 ± 0.33% and 52.12 ± 0.78% for MCF-7 and HEpG2 cells, respectively (Fig. 6). The effect produced by 6-Gin + Dox-m-HAP is significant (0.05 < P) which could arise due to the synergistic effect of 6-gingerol increasing the anti-proliferative effect of doxorubicin [32]. However, it demonstrated that the cell viability results further represent the findings of cytotoxicity assays and fluorescence imaging studies.

### Cytotoxic effects on non-cancerous Vero cells

**Fig. 7:**
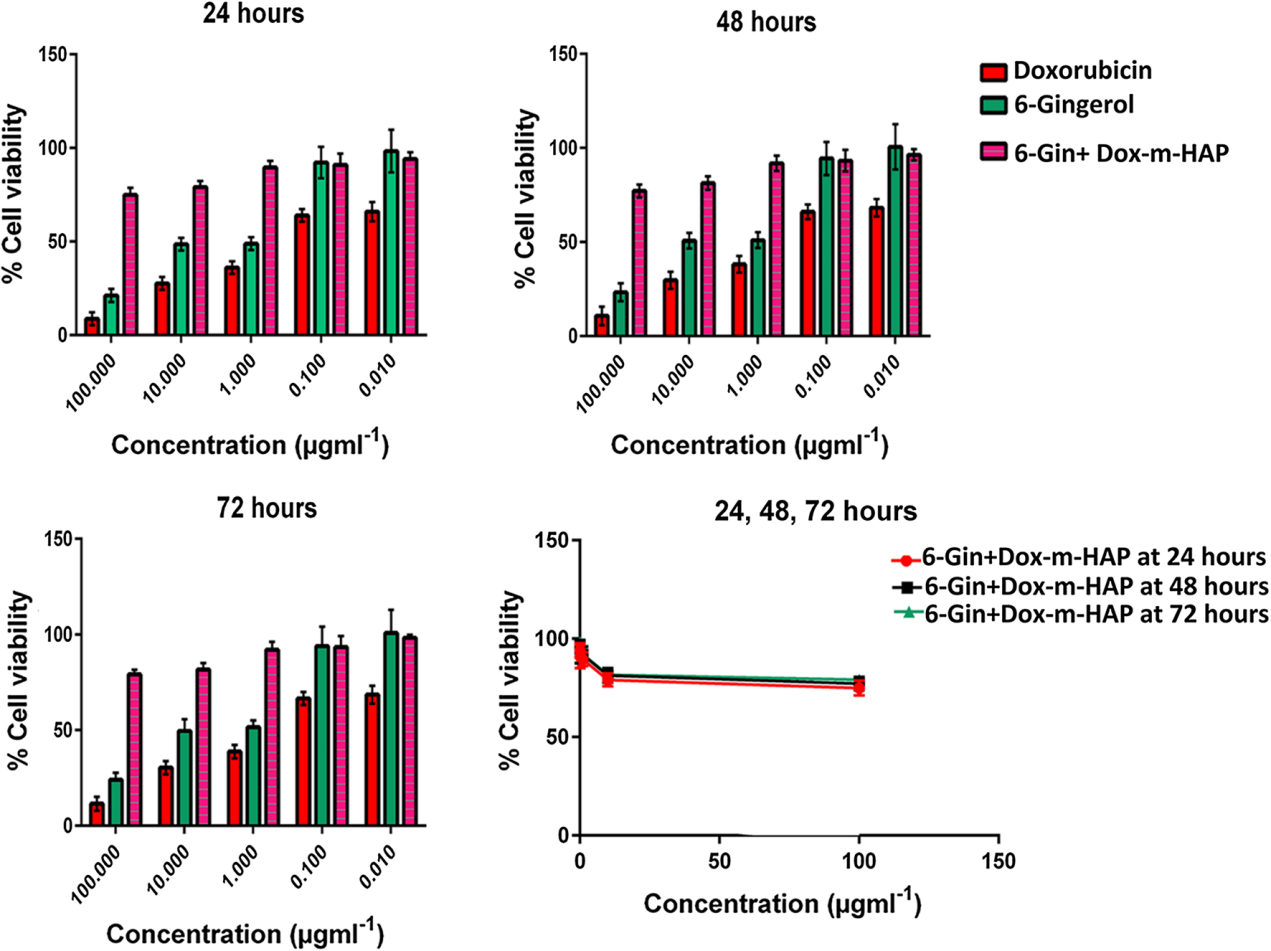
Cell viability of Vero cells after treating with doxorubicin, 6-gingerol and 6-Gin + Dox-m-HAP for 24, 48 and 72 h

According to the dose response curves given in Additional file 1: Fig. S10a, b, it is clear that the free 6-gingerol and doxorubicin have produced very high toxicity on Vero cells and that this effect has been drastically reduced when these drugs have been incorporated into the m-HAP nanoparticles. This effect is much more evident with the increase in the concentration of the drug loaded nanoparticles. And importantly they have maintained a higher cell viability in the range of concentration that has been effective against the MCF-7 cells and HEpG2 cells. Further, it is evident that when the drugs are co-fabricated, as 6-Gin + Dox-m-HAP (Fig. 7), toxicity is considerably reduced compared to the singly loaded systems. All these results suggest that these drug loaded nanoparticles produce more effects selectively on cancer cells, while minimizing the effects on non-cancerous cells.

## Conclusions

In this work we have successfully prepared 6-Gin-m-HAP, Dox-m-HAP and 6-Gin + Dox-m-HAP for the targeted and controlled release of 6-gingerol and doxorubicin and their combinations in a pH sensitive manner. The TEM results and surface characteristics provided by FT-IR and XPS analysis confirmed the interaction of drug molecules with m-HAP. The slow release of the drug molecules, preferably in a low pH environment (pH 5.3), further exhibited the pH responsiveness at the sites of the cancer cells. Furthermore, it has been shown that 6-Gin-m-HAP, Dox-m-HAP and co-loaded 6-Gin + Dox-m-HAP have a potent inhibitory effect on both breast and liver cancer cells to a greater extent than the free doxorubicin and free 6-gingerol molecules. The cell proliferation assays conducted on MCF-7 cells and HEpG2 cells suggested that the anti-cancer effect of 6-gingerol is much enhanced when incorporated into the nanocarrier system. Additionally, the results obtained from the cell proliferation assays, fluorescence imaging and flow cytometric analysis, showed that the combinational approach of both 6-gingerol and doxorubicin in m-HAP has demonstrated much enhanced activity over MCF-7 and HEpG2 cancer cells, suggesting the chemosensitive activity of 6-gingerol on doxorubicin. And this effect could be identified as beneficial, as it could reduce the amount of doxorubicin used and thereby its associated toxic effects. Additionally, cell proliferation detection assays carried out on Vero cells highlighted that these drug loaded nanoparticles would have no or low cytotoxicity on non-targeted cells. Therefore, this work highlights the possibility of developing new drug carrier systems for the effective delivery of anti-cancer agents like doxorubicin together with 6-gingerol like chemo preventive agents, to induce higher anti-proliferative activity while minimizing the drawbacks observed with doxorubicin.

## Additional file

**Additional file 1.** XPS analysis, cumulative drug release percentages, dose responsive and time response curves and flow cytometry data.

## Abbreviations

m-HAP: magnetically active hydroxyapatite
Dox-m-HAP: doxorubicin loaded m-HAP
6-Gin-m-HAP: 6-gingerol in m-HAP
6-Gin + Dox-m-HAP: 6-gingerol and doxorubicin in m-HAP
ANX: Annexin V
GR: Zombie green
