# Supplementary material for: Fabrication of 6-gingerol, doxorubicin and alginate hydroxyapatite into a bio-compatible formulation: enhanced anti-proliferative effect on breast and liver cancer cells
Source: Chem Cent J. 2018 Nov 23;12:119. doi: 10.1186/s13065-018-0482-6 (PMC6768026; doi:10.1186/s13065-018-0482-6)
Supplement: Supplementary file 1 — Additional file 1. XPS analysis, cumulative drug release percentages, dose responsive and time response curves and flow cytometry data. [file 13065_2018_482_MOESM1_ESM.docx]

**Fabrication of 6-gingerol, doxorubicin and alginate hydroxyapatite in to a bio-compatible formulation: Enhanced anti-proliferative effect on breast and liver cancer cells**

***Danushika C. Manatunga,^a^ Rohini M. de Silva,^a*^ K. M. Nalin de Silva,^a,b^ Dulharie T. Wijeratne,^c^ Gathsaurie Neelika Malavige,^c^ Gareth Williams^d^***

**SUPPLEMENTARY INFORMATION**


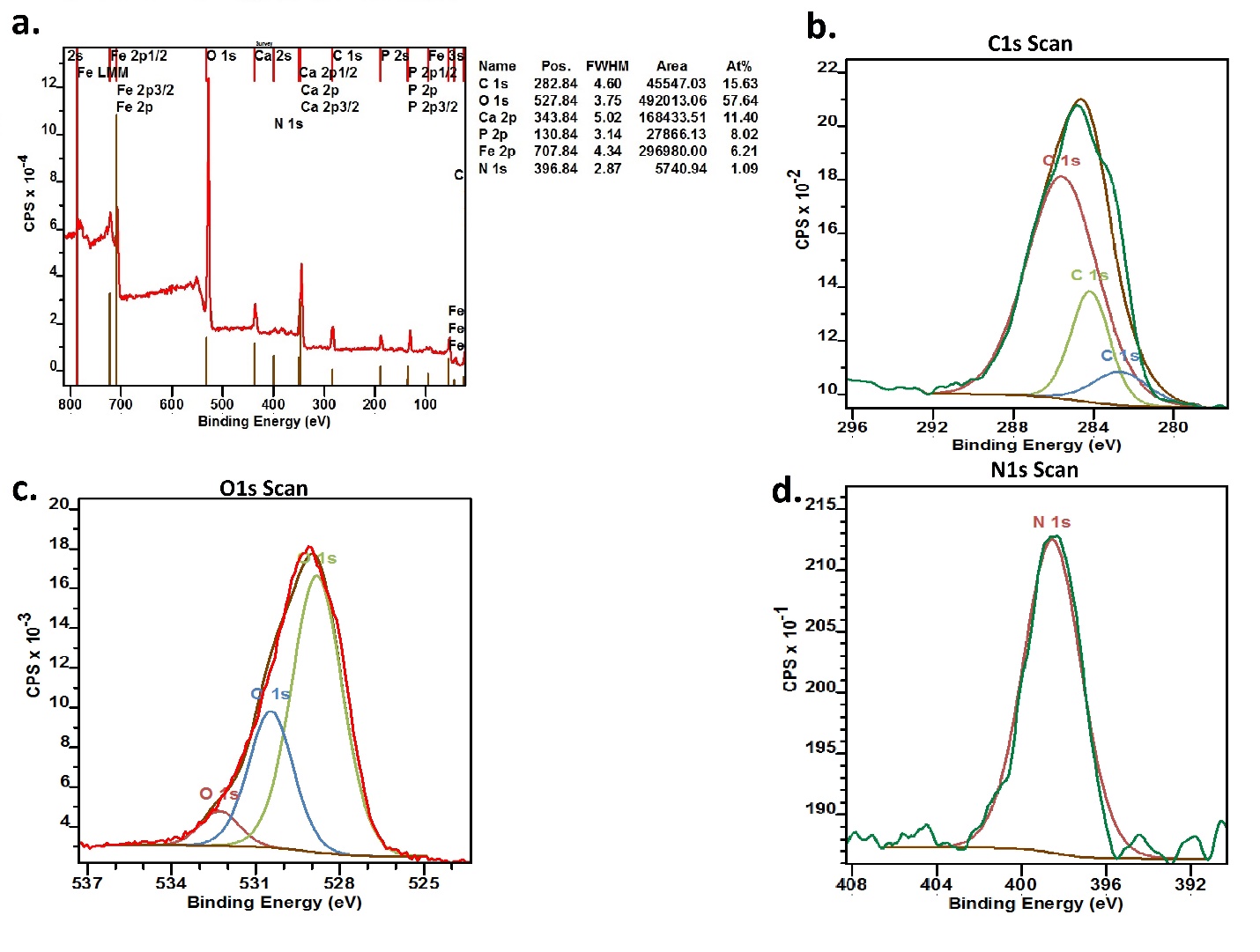


Fig. S1. XPS analysis of m-HAP system a) Different elemental compositions with respective percentages, b) Binding energy spectrum of C1s, c) O1s, d) N1s

- Percentage of each element

carbon = 5.63%, % of oxygen = 57.64%, % of nitrogen= 1.09%, percentage of Fe= 6.21%, percentage of Ca= 11.40%

- C1s binding energy of 285.5769 eV would result from (C-O-C) of alginate [1] and it has shifted from 285.8 eV with the binding in to HAp and IONPs.
- 284.1693 eV and 282.6416 eV will arise from the –C-C- and –C-H groups of the polymer backbone as observed in previous work [2],[3], but it has shifted to lower binding energy values with the interaction to HAp and IONPs.
- The O1s binding energy of 532.2 eV would appear due to –COO^-^ groups (an expected value of 532.3 eV) in the alginate backbone [1],[4]. Additionally, it will also highlight the the presence of –OH of adsorbed water (an expected value of 532.8 eV).
- O1s peak at 528.8093 eV could be due to the Fe-O bond (an expected value of 530.1 eV) [5] and little changes may suggest the coordination of the –Fe with the polymer molecules[4].
- O1s binding energy value of 530.4555 eV can be expected from the oxygen of HAp with a shift due to the possible interactions [5],[6],[7],[8].
- In N 1s spectrum, a weakly interacted ammonia[9] would give rise to a peak at 398.5448 eV.


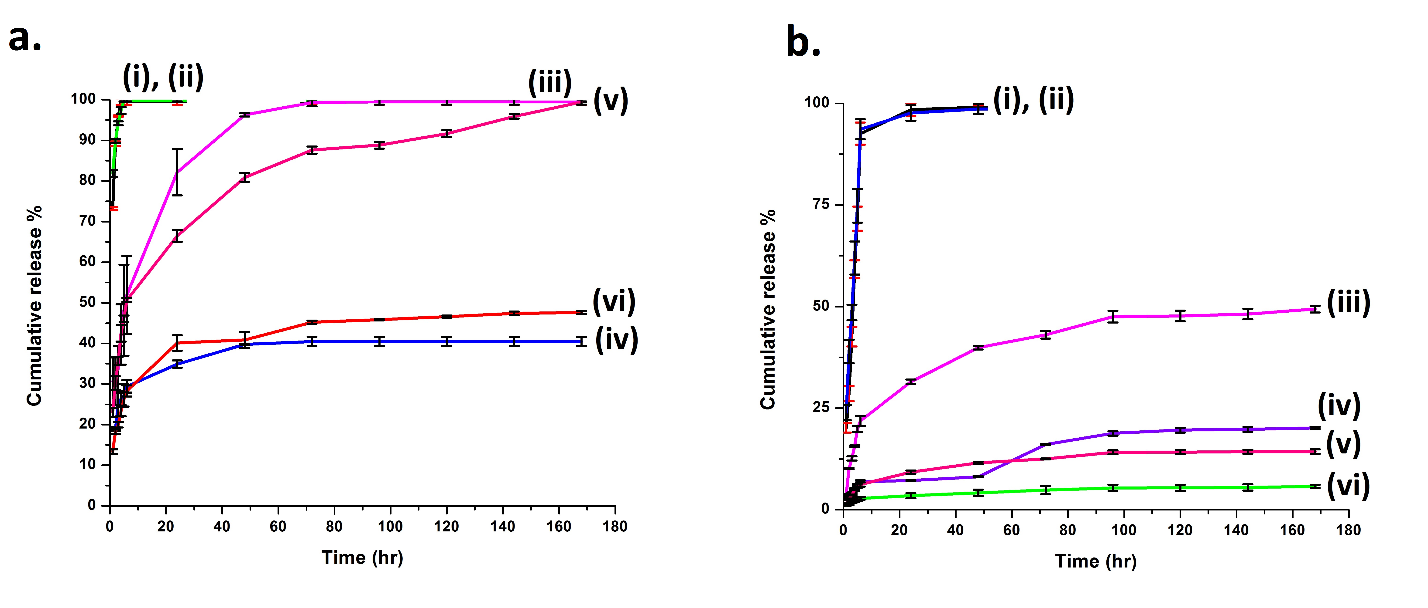
Fig. S2. Cumulative drug release percentage of a) neat 6-gingerol via the dialysis membrane at pH 5.3 (i), neat 6-gingerol at pH 7.4 (ii), 6-gingerol release from 6-Gin-m-HAP at pH 5.3 (iii), 6-gingerol release from 6-Gin-m-HAP at pH 7.4 (iv), 6-gingerol release from 6-Gin+Dox-m-HAP at pH 5.3 (v), 6-gingerol release from 6-Gin+Dox-m-HAP at pH 7.4 (vi), b) release of neat doxorubicin via the dialysis membrane at pH 5.3 (i), release of neat doxorubicin via the dialysis membrane at pH 7.4 (ii), doxorubicin release from Dox-m-HAP at pH 5.3 (iii), doxorubicin release from Dox-m-HAP at pH 7.4 (iv), doxorubicin release from 6-Gin+ Dox-m-HAP at pH 5.3 (v), doxorubicin release from 6-Gin+ Dox-m-HAP at pH 7.4 (vi). Results are presented as mean ± SD, n = 3.


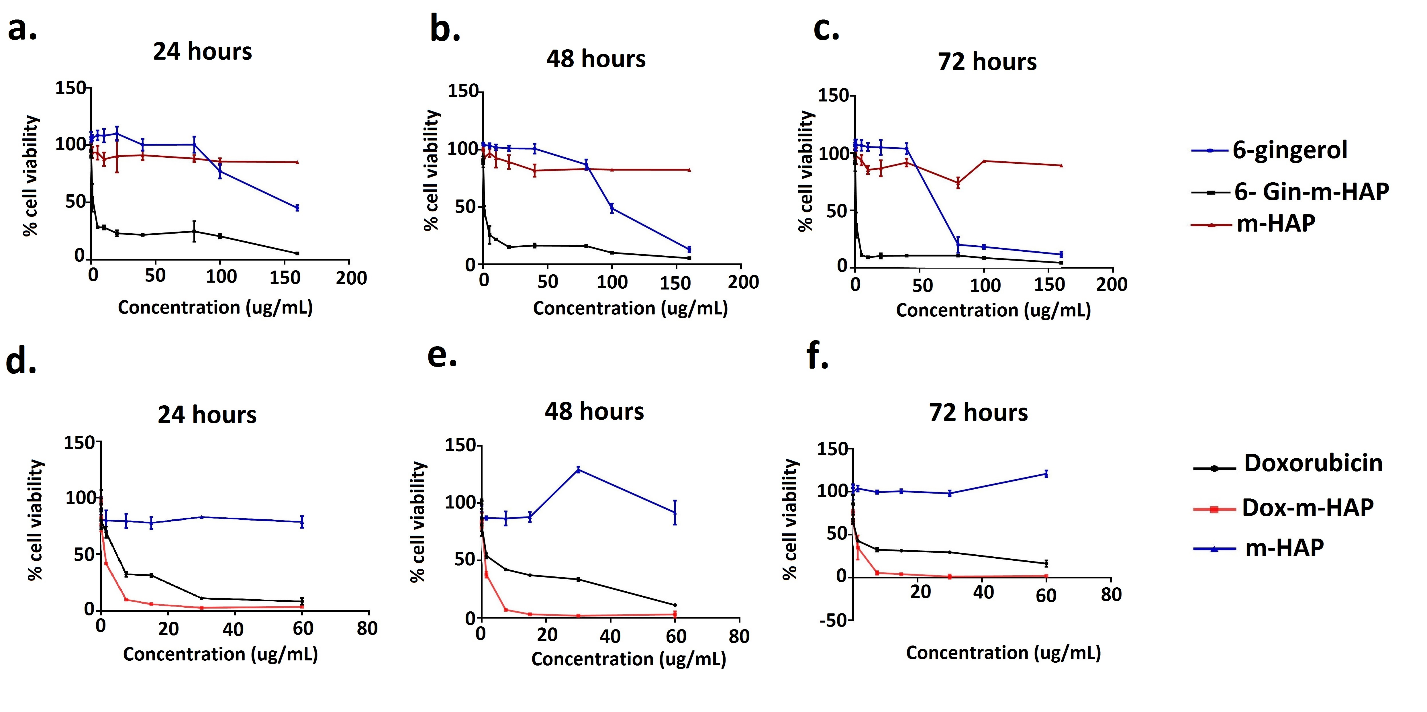


Fig. S3. Dose responsive and time response curves of MCF 7 cells treated with a- c) 6-gingerol, 6-Gin-m-HAP and m-HAP for 24-72 hours, d- f) a) MCF-7 cells treated with doxorubicin, Dox-m-HAP and m-HAP for 24-72 hours. Results are presented as mean ± SD, from three independent experiments each containing three replicates.


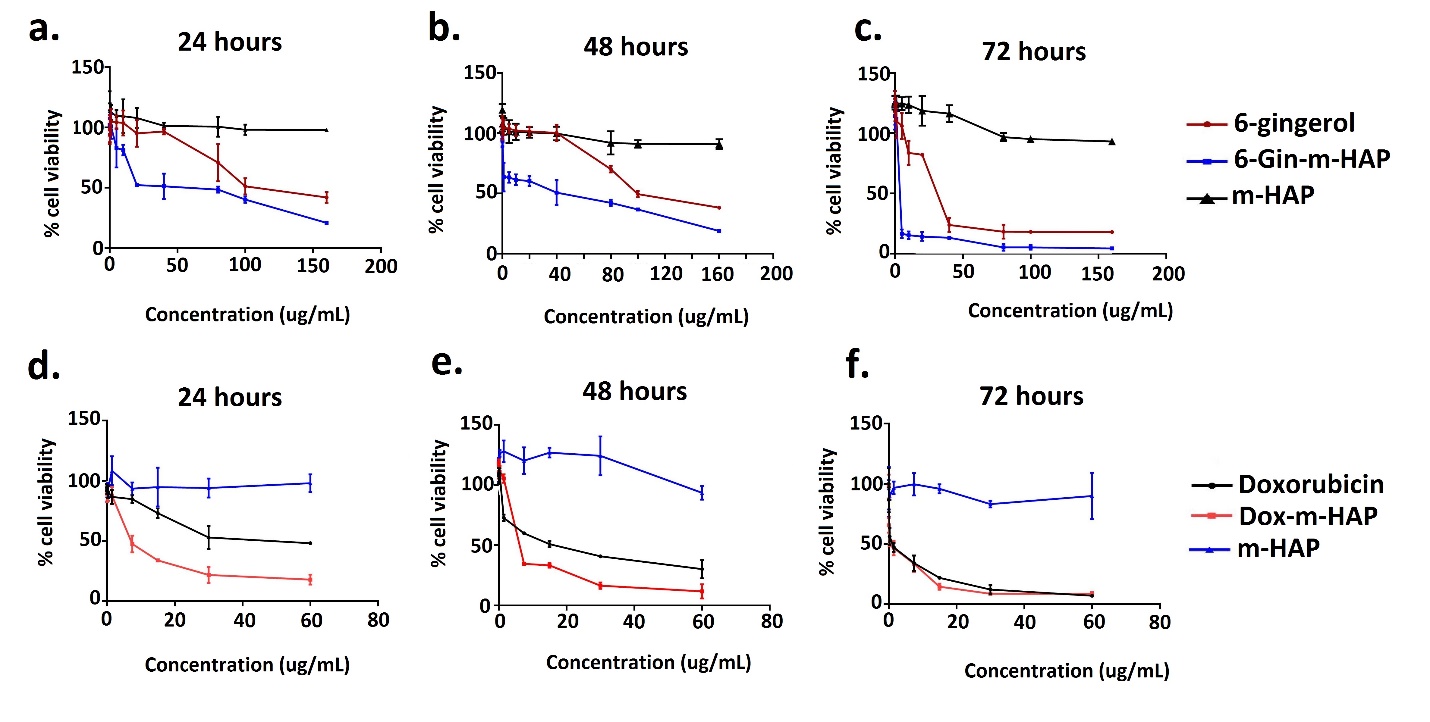


Fig. S4. Dose responsive and time response curves of HEpG2 cells treated with a- c) 6-gingerol, 6-Gin-m-HAP and m-HAP for 24-72 hours, d- f) a) HEpG2 cells treated with doxorubicin, Dox-m-HAP and m-HAP for 24-72 hours. Results are presented as mean ± SD, from three independent experiments each containing three replicates.


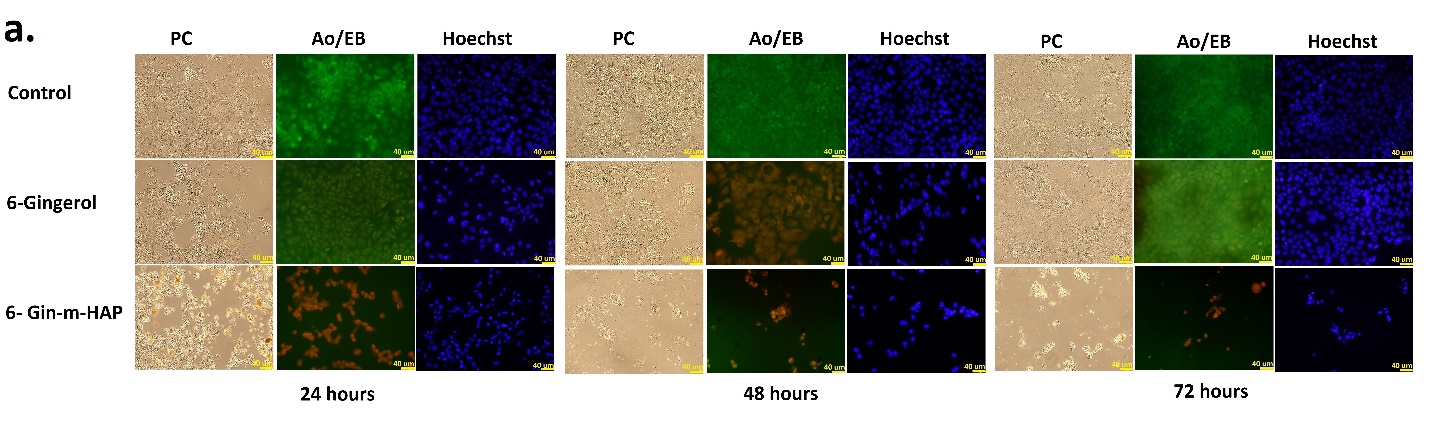


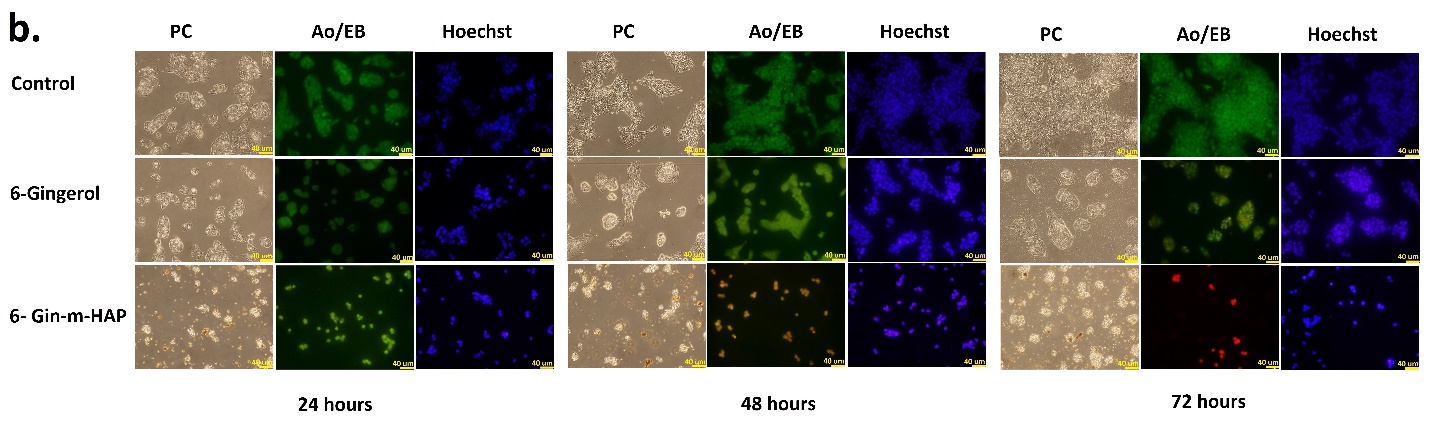


Fig. S5. Phase contrast (PC) and fluorescence images obtained to assess the effect of 6-gingerol and 6-Gin-m-HAP on a) MCF-7 cells and b) HEpG2 cells over 24-72 hours


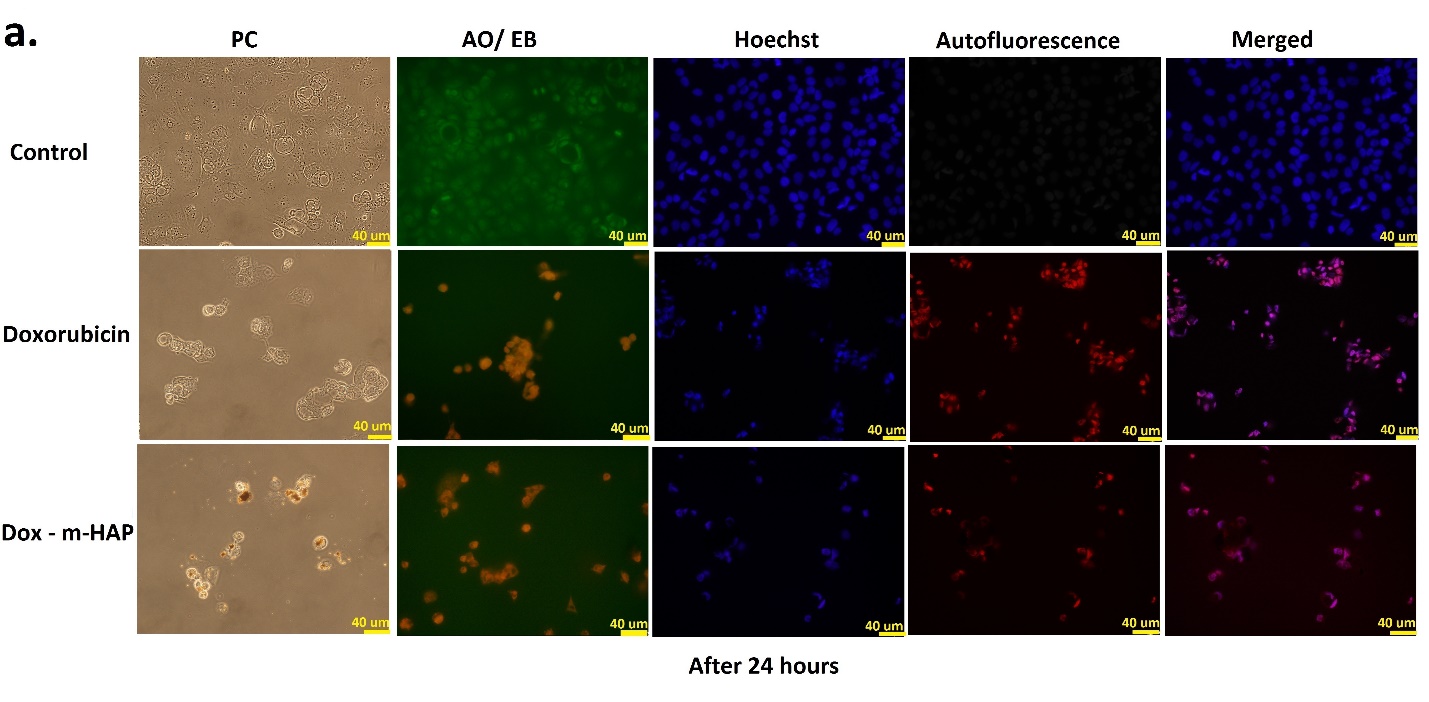


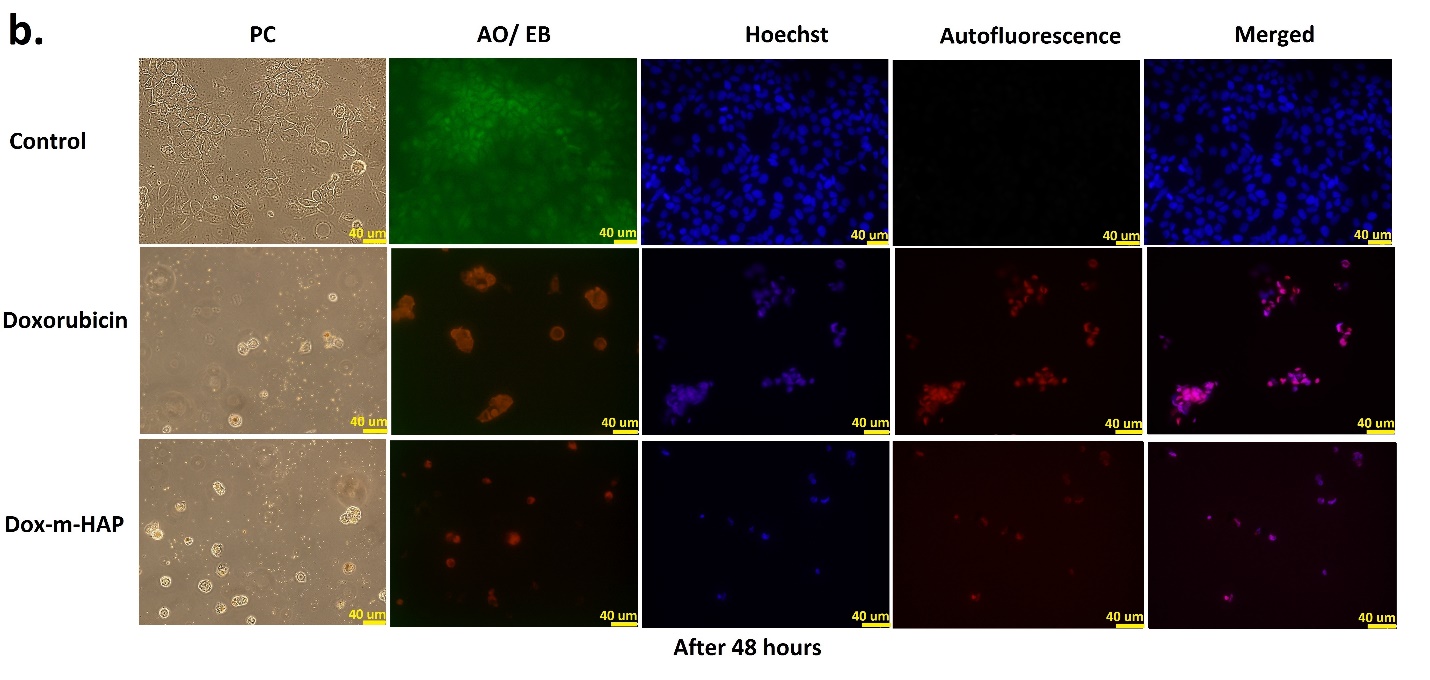


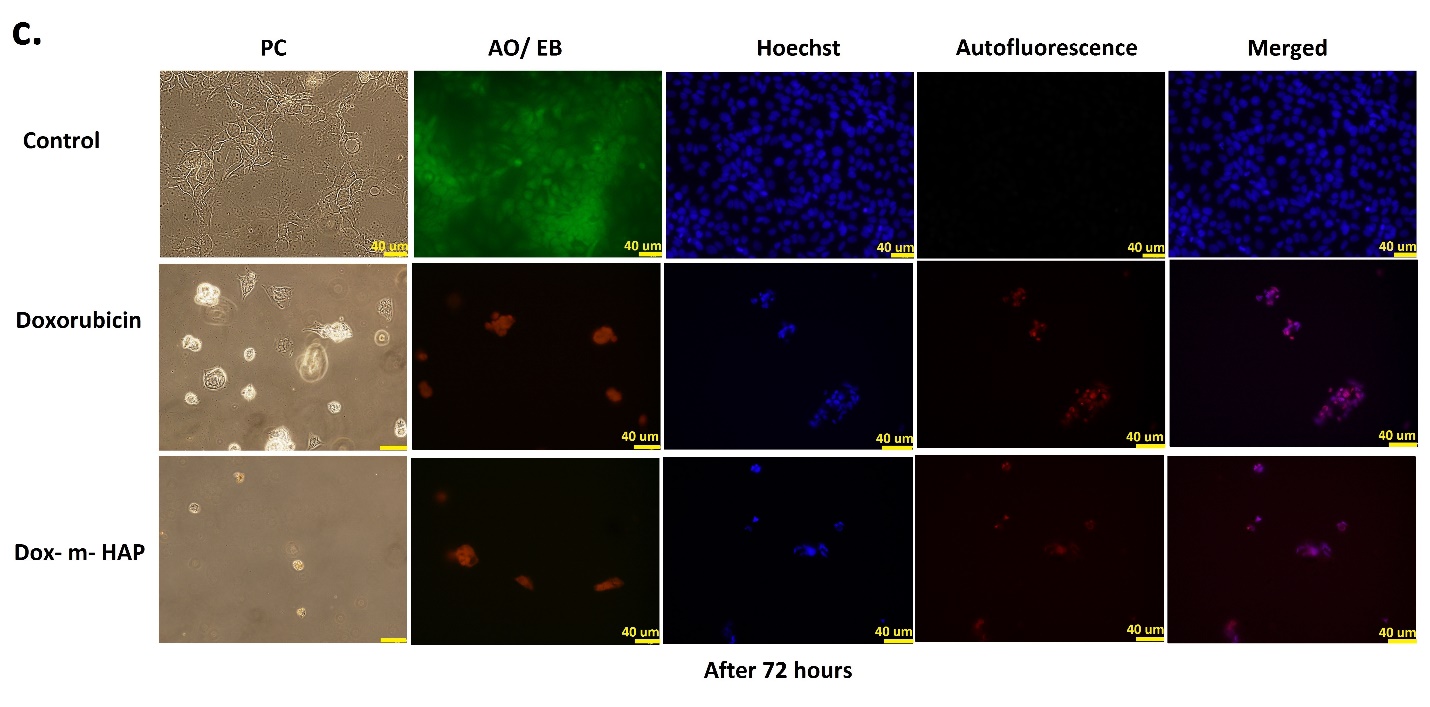


Fig. S6. Phase contrast (PC) and fluorescence images obtained to assess the effect of doxorubicin and Dox-m-HAP on MCF-7 cells over a) 24 hours, b) 48 hours and c) 72 hours


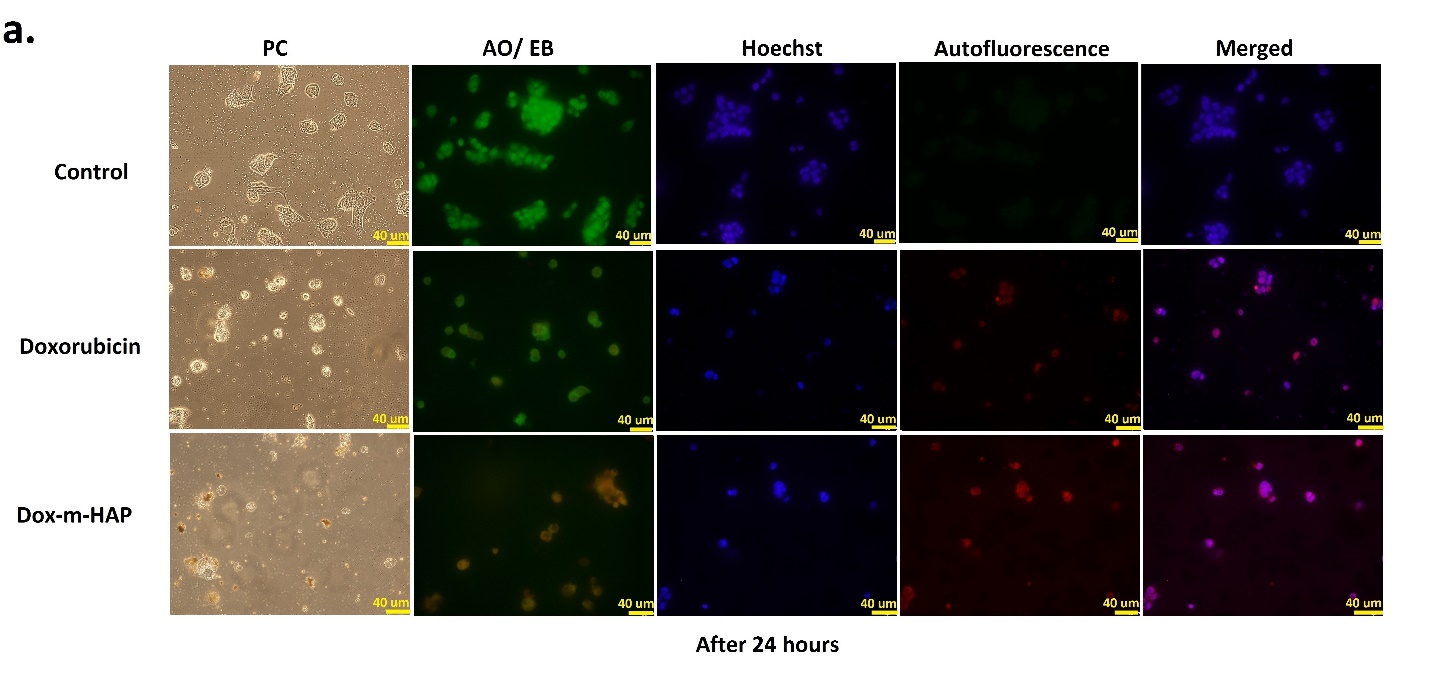


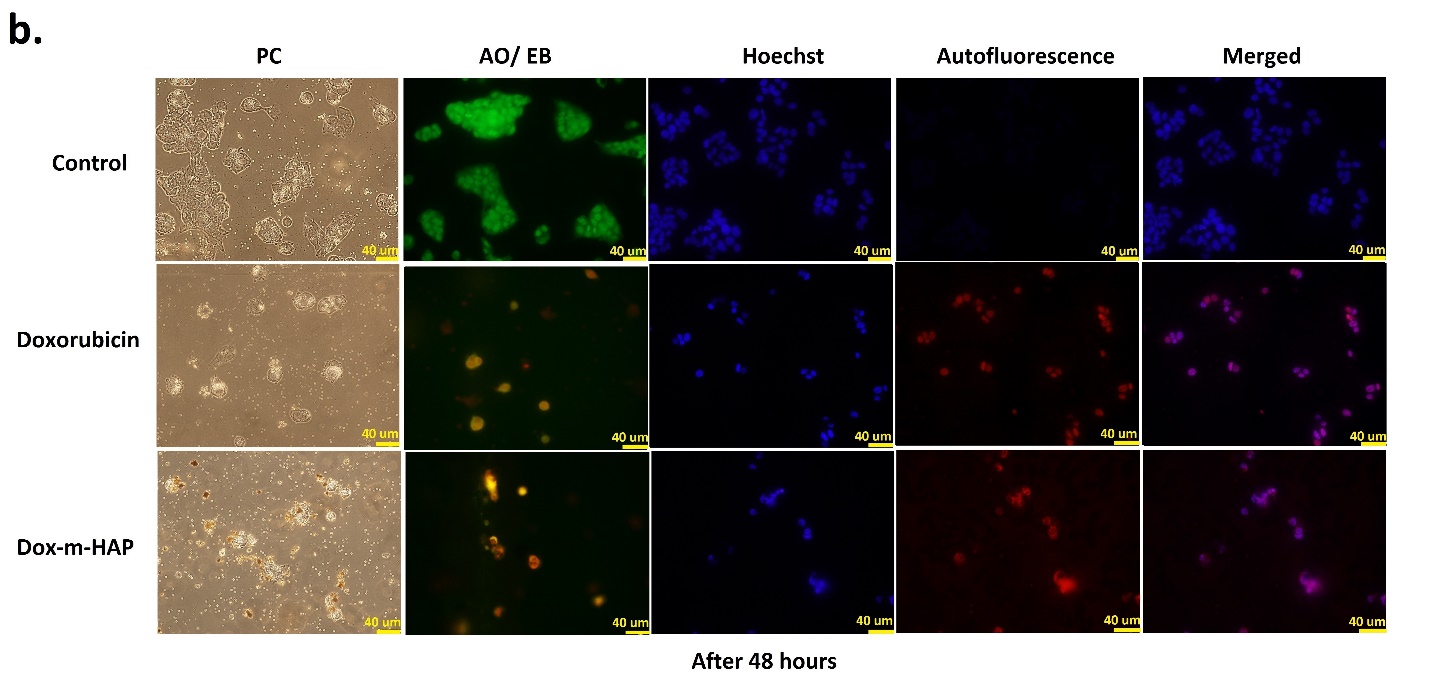


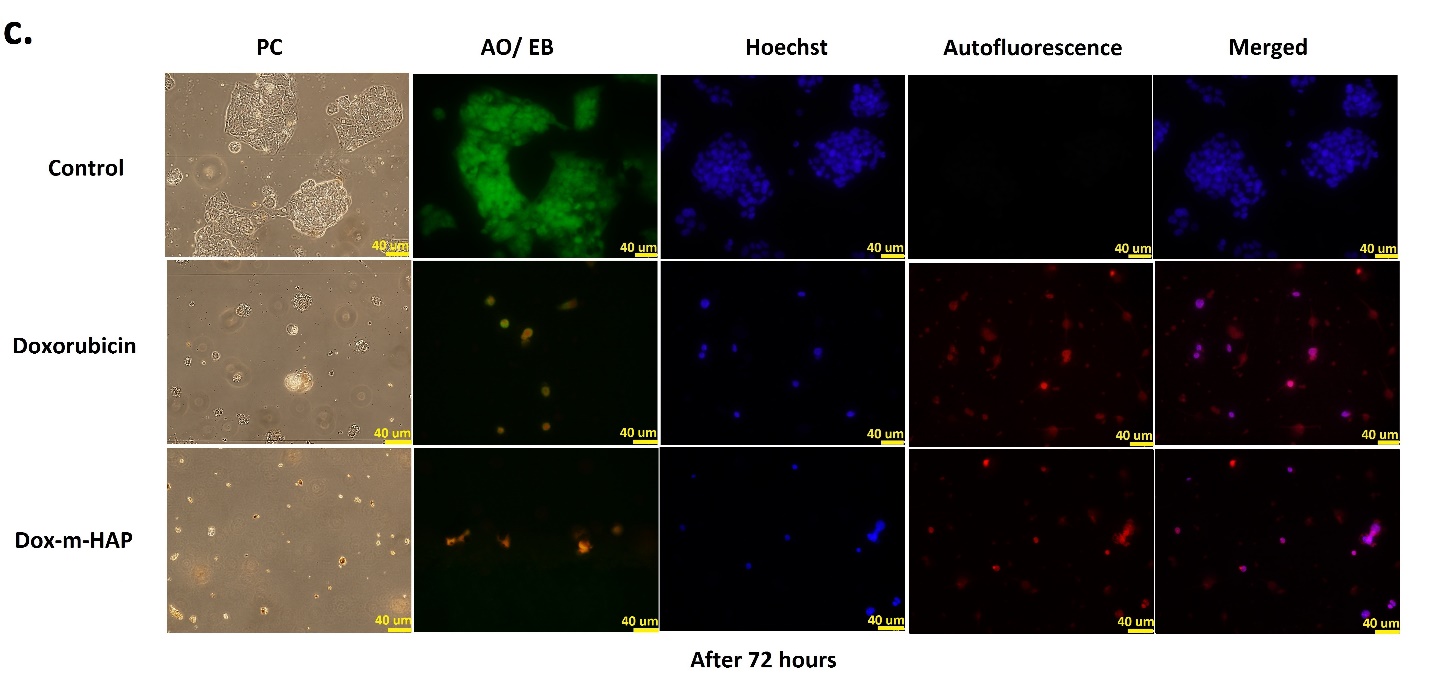


Fig. S7. Phase contrast (PC) and fluorescence images obtained to assess the effect of doxorubicin and Dox-m-HAP on HEpG2 cells over a) 24 hours, b) 48 hours and c) 72 hours


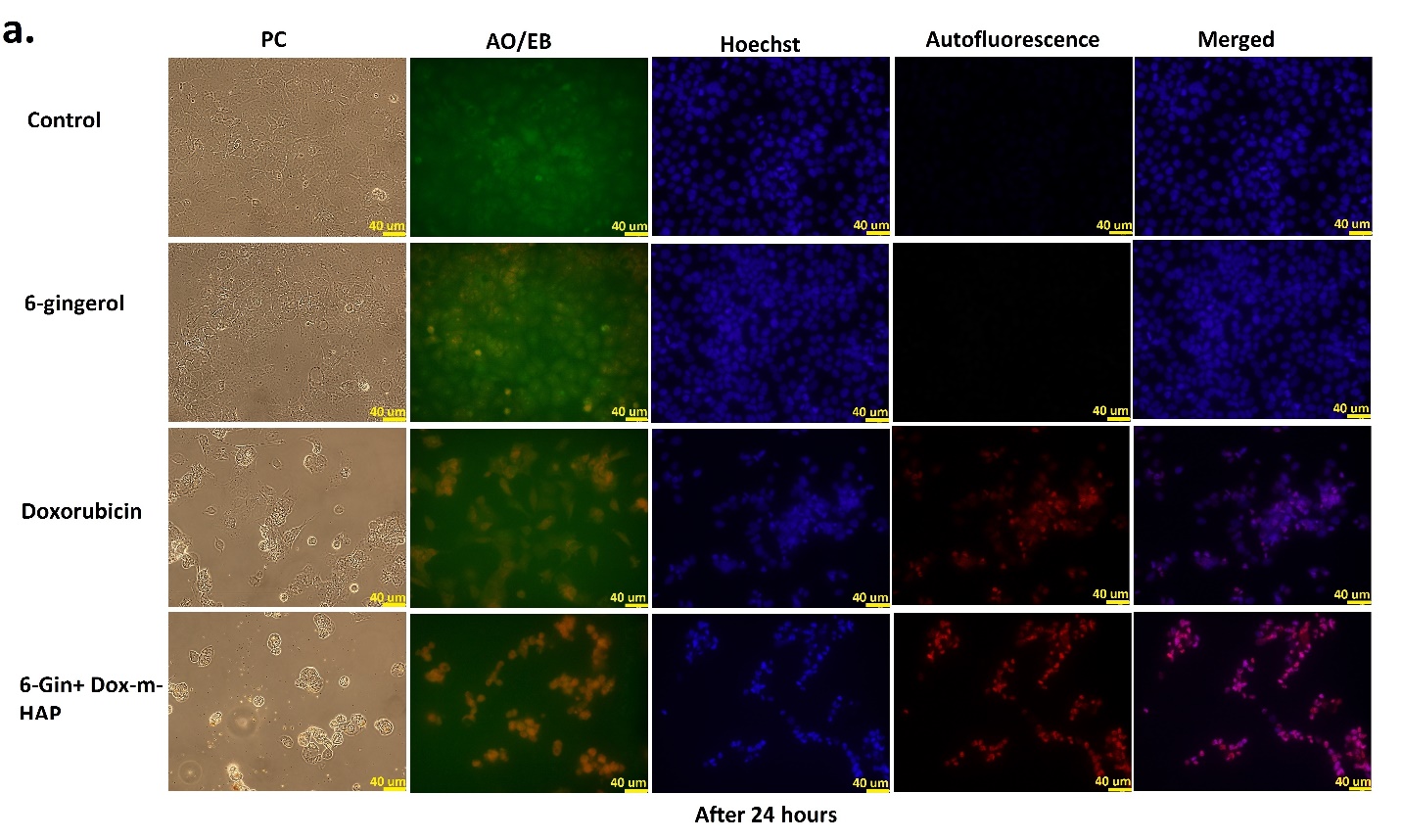


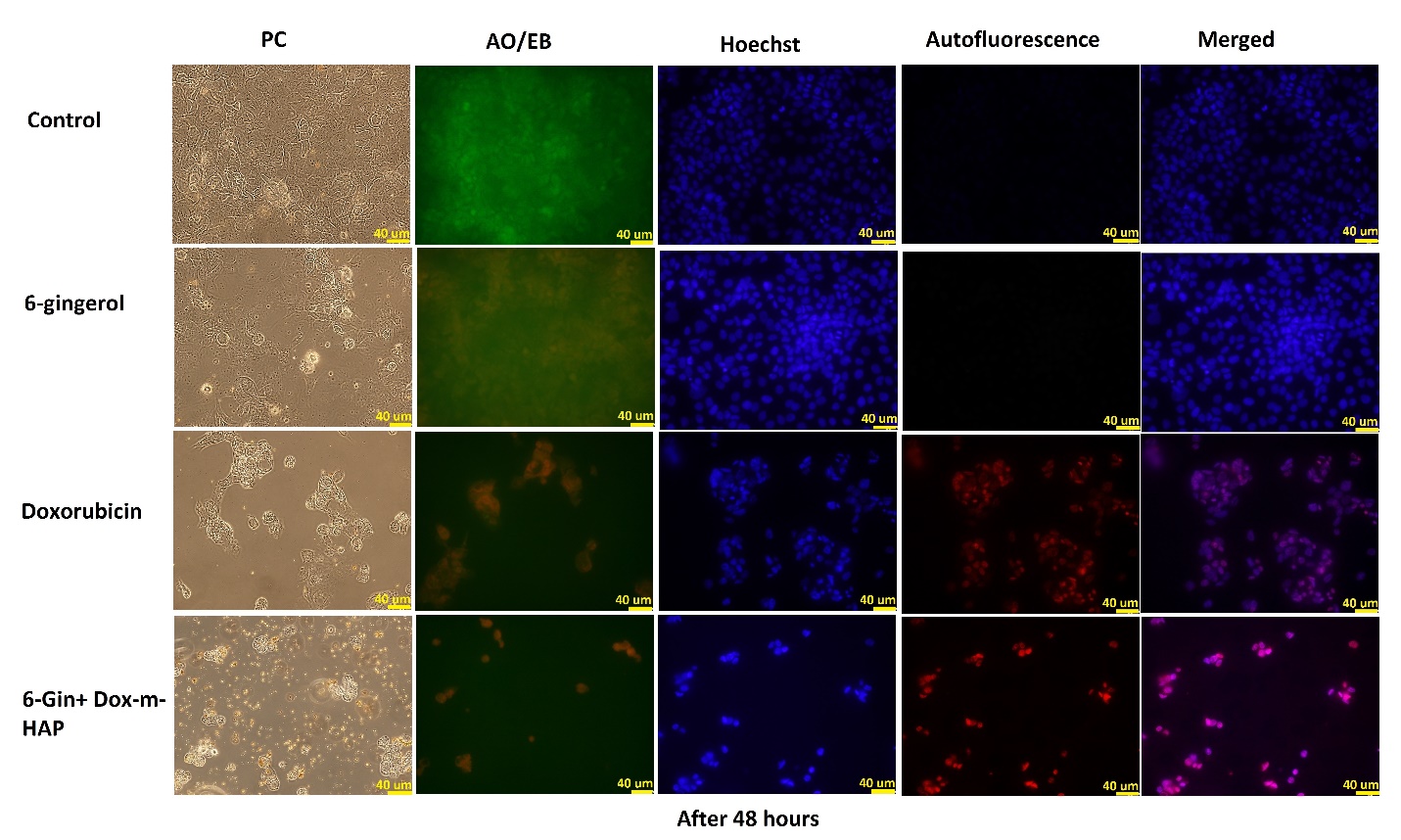


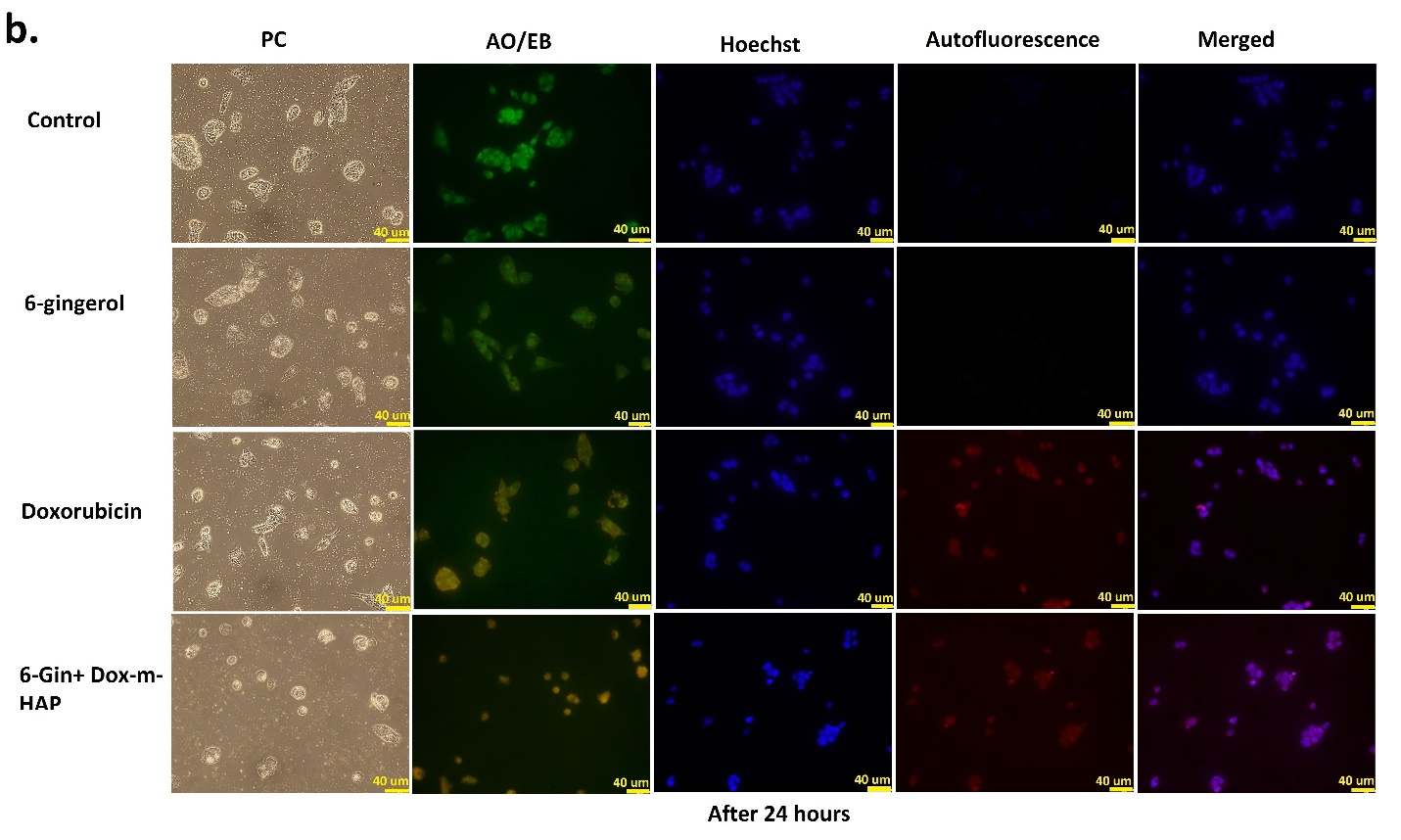


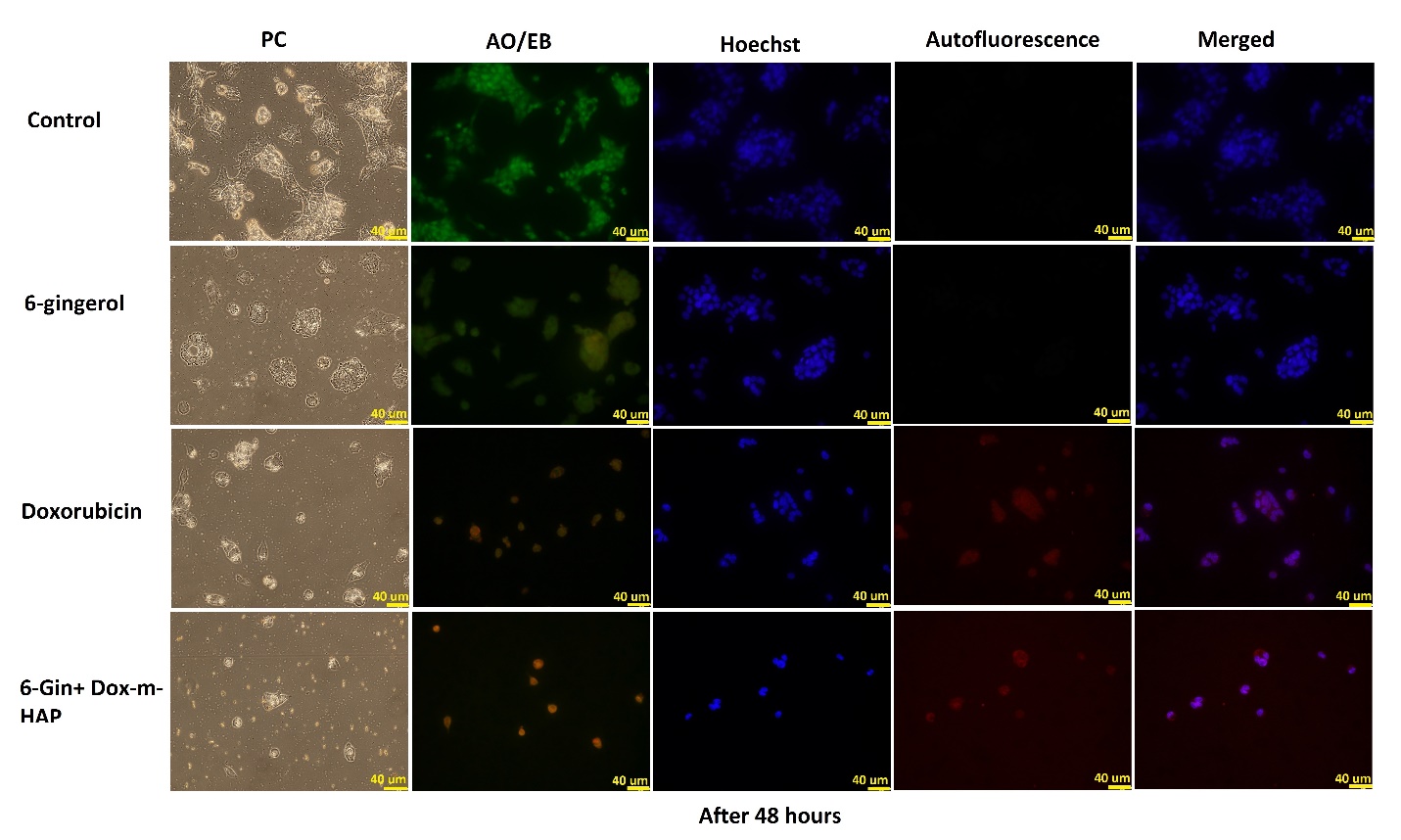


Fig. S8. Phase contrast (PC) and fluorescence images obtained to assess the effect of 6-Gin+ Dox-m-HAP on a) MCF-7 cells for 24 and 48 hours and b) HEpG2 cells for 24 and 48 hours


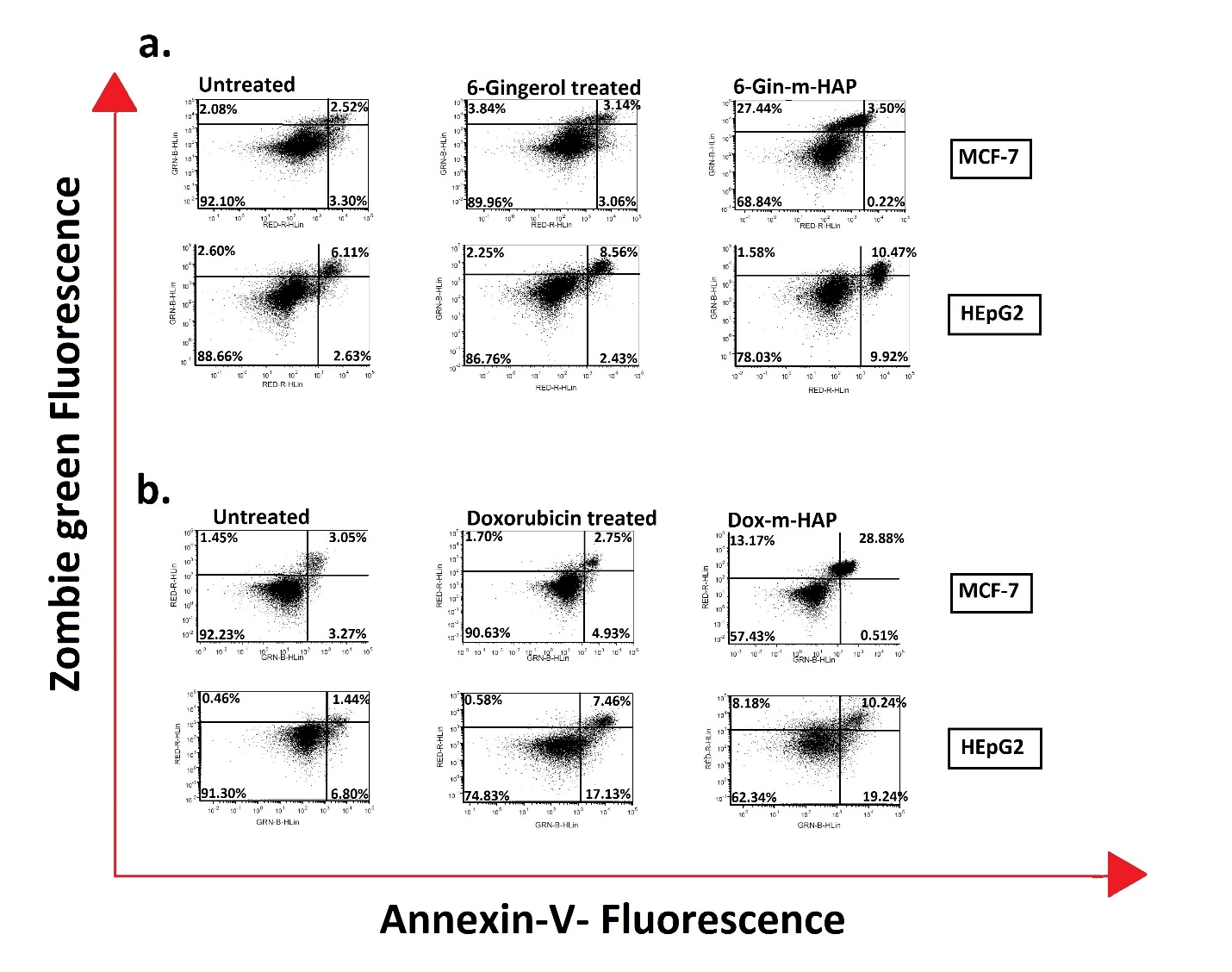


Fig. S9. Flow cytometric analysis of apoptotic induction of MCF-7 and HEpG2 cells by 6-gingerol, doxorubicin, 6-Gin-m-HAP and 6-Gin+Dox-m-HAP after staining with Annexin V (ANX) and Zombie green (ZGR) dyes. ANX^-^/ZGR^+^ - necrotic or debris cells; ANX^+^/ZGR^+^- late apoptotic cells; ANX^-^/ZGR low- viable; ANX^+^/ZGR dim- apoptotic cells. Numbers in each quadrant represent the percentage of cells (data are representative of mean ± SD of triplicated of three individual experiments)


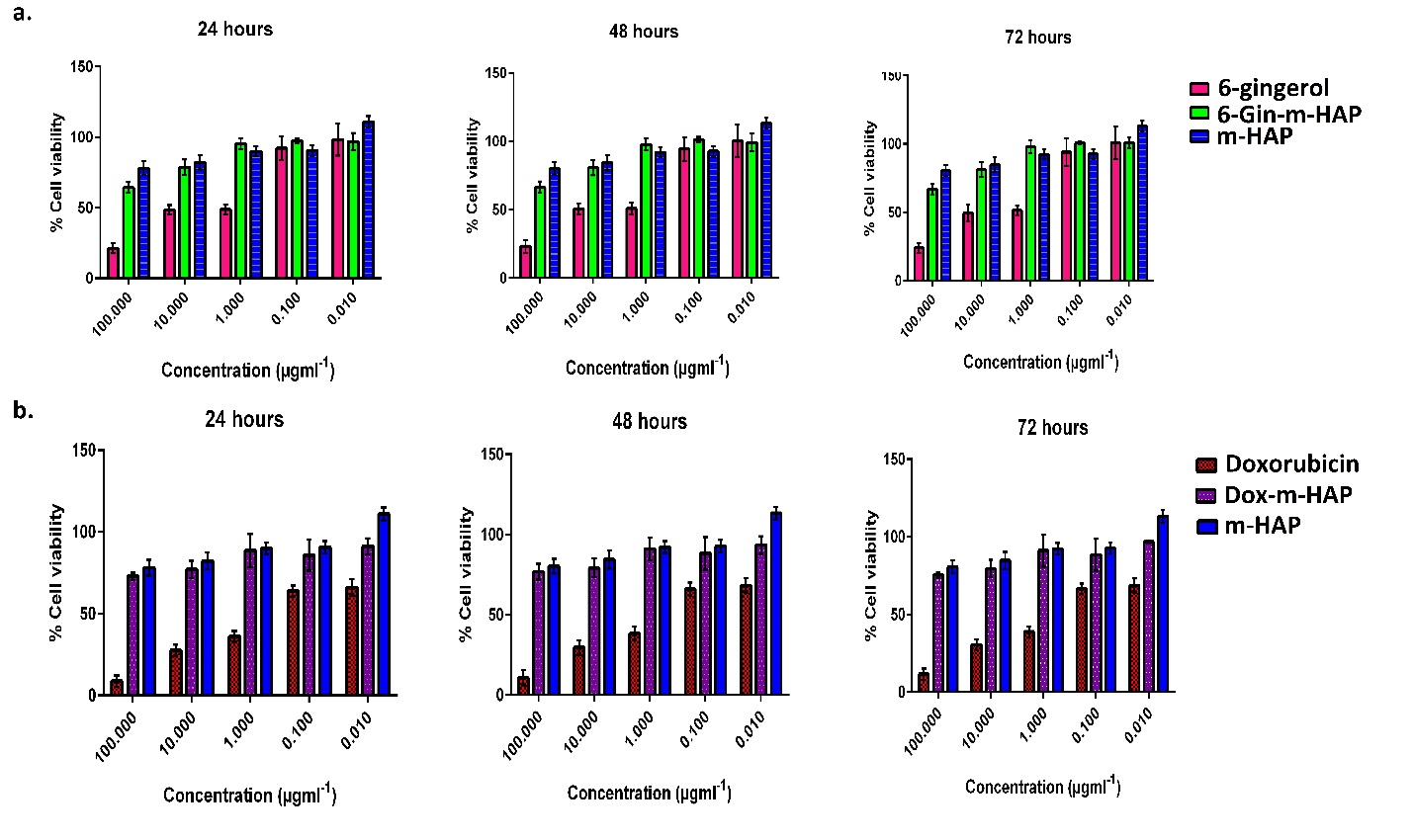


Fig. S10. Cell viability of Vero cells in the presence of a) 6-gingerol, 6-Gin-m-HAP, m-HAP and b) doxorubicin, Dox-m-HAP and m-HAP (Data are representative of mean± SD from three individual experiments, each having three replicates)

1. Chen JP, Hong L, Wu S, Wang L (2002) Elucidation of interactions between metal ions and Ca alginate-based ion-exchange resin by spectroscopic analysis and modeling simulation. Langmuir 18:9413–9421 . doi: 10.1021/la026060v

2. Bhattacharya S, Mallik D, Nayar S (2011) Comparative study of biomimetic iron oxides synthesized using microwave induced and conventional method. IEEE Trans Magn 47:1647–1652 . doi: 10.1109/TMAG.2011.2104418

3. Lei H, Wei Q, Wang Q, et al (2017) Characterization of ginger essential oil / palygorskite composite ( GEO-PGS ) and its anti-bacteria activity. MSC 73:381–387 . doi: 10.1016/j.msec.2016.12.093

4. Li B, Dong Y, Zou C, Xu Y (2014) Iron(III)-alginate fiber complex as a highly effective and stable heterogeneous fenton photocatalyst for mineralization of organic dye. Ind Eng Chem Res 53:4199–4206 . doi: 10.1021/ie404241r

5. Mercado DF, Magnacca G, Malandrino M, et al (2014) Paramagnetic iron-doped hydroxyapatite nanoparticles with improved metal sorption properties. A bioorganic substrates-mediated synthesis. ACS Appl Mater Interfaces 6:3937–3946 . doi: 10.1021/am405217j

6. Costescu A, Pasuk I, Ungureanu F, et al (2010) Physico-Chemical Properties Of Nano-Sized Hexagonal Hydroxyapatite Powder Synthesized By Sol-Gel. Dig J Nanomater Biostructures 5:989–1000

7. Lu HB, Campbell CT, Graham DJ, Ratner BD (2000) Surface characterization of hydroxyapatite and related calcium phosphates by XPS and TOF-SIMS. Anal Chem 72:2886–2894 . doi: 10.1021/ac990812h

8. Mercado DF, Rubert A, Magnacca G, et al (2017) Versatile Fe-Containing Hydroxyapatite Nanomaterials as Efficient Substrates for Lead Ions Adsorption. J Nanosci Technol 17:1–10 . doi: 10.1166/jnn.2017.13870

9. Strongin D, Mowlem J (1991) A NEXAFS study on the adsorption of ammonia on clean and potassium-promoted iron. Surf Sci Lett 253: L209- L214. doi: 10.1016/0167-2584(91)90370-7
